# Characterizing metabolic stress-induced phenotypes of *Synechocystis* PCC6803 with Raman spectroscopy

**DOI:** 10.7717/peerj.8535

**Published:** 2020-03-30

**Authors:** Imen Tanniche, Eva Collakova, Cynthia Denbow, Ryan S. Senger

**Affiliations:** 1 Department of Biological Systems Engineering, Virginia Polytechnic Institute and State University (Virginia Tech), Blacksburg, VA, United States of America; 2 School of Plant & Environmental Sciences, Virginia Polytechnic Institute and State University (Virginia Tech), Blacksburg, VA, United States of America; 3 Department of Chemical Engineering, Virginia Polytechnic Institute and State University (Virginia Tech), Blacksburg, VA, United States of America

**Keywords:** Raman spectroscopy, Cyanobacteria, *Synechocystis*, Microbial phenotyping, Rametrix™, Principal component analysis, Discriminant analysis

## Abstract

**Background:**

During their long evolution, *Synechocystis* sp. PCC6803 developed a remarkable capacity to acclimate to diverse environmental conditions. In this study, Raman spectroscopy and Raman chemometrics tools (Rametrix^TM^) were employed to investigate the phenotypic changes in response to external stressors and correlate specific Raman bands with their corresponding biomolecules determined with widely used analytical methods.

**Methods:**

*Synechocystis* cells were grown in the presence of (i) acetate (7.5–30 mM), (ii) NaCl (50–150 mM) and (iii) limiting levels of MgSO_4_ (0–62.5 mM) in BG-11 media. Principal component analysis (PCA) and discriminant analysis of PCs (DAPC) were performed with the Rametrix^TM^ LITE Toolbox for MATLAB^Ⓡ^. Next, validation of these models was realized via Rametrix^TM^ PRO Toolbox where prediction of accuracy, sensitivity, and specificity for an unknown Raman spectrum was calculated. These analyses were coupled with statistical tests (ANOVA and pairwise comparison) to determine statistically significant changes in the phenotypic responses. Finally, amino acid and fatty acid levels were measured with well-established analytical methods. The obtained data were correlated with previously established Raman bands assigned to these biomolecules.

**Results:**

Distinguishable clusters representative of phenotypic responses were observed based on the external stimuli (i.e., acetate, NaCl, MgSO_4_, and controls grown on BG-11 medium) or its concentration when analyzing separately. For all these cases, Rametrix^TM^ PRO was able to predict efficiently the corresponding concentration in the culture media for an unknown Raman spectra with accuracy, sensitivity and specificity exceeding random chance. Finally, correlations (*R* > 0.7) were observed for all amino acids and fatty acids between well-established analytical methods and Raman bands.

## Introduction

Microbial cells undergo phenotypic variations in response to changing environmental conditions. These changes directly affect the chemical composition of the cell, allowing it to maintain integrity, and the study of these changes is referred to as “microbial phenotyping” (Tummala et al., 2003; Sellick & Reece, 2005; Alsaker, Paredes & Papoutsakis, 2010; Nicolaou, Gaida & Papoutsakis, 2010). For example, exposure to high salt concentrations (e.g., NaCl, MgSO_4_) results in ionic and osmotic imbalances (Markovitz & Sylvan, 1962; Lusk, Williams & Kennedy, 1968; Reed et al., 1985; Mikkat et al., 2000; Karandashova & Elanskaya, 2005; Marin et al., 2006). This leads to the accumulation of carboxylic acids (e.g. acetate), which can be toxic and require a reorganization of the cell membrane for survival (Papoutsakis et al., 1987; Russell, 1992; Lasko et al., 1997; Roe et al., 2002). Thus, cells have the ability to respond to such chemical stresses, adapt for survival, and even acquire tolerance (Nicolaou, Gaida & Papoutsakis, 2010). These characteristics are necessary in biotechnology, where microbes are engineered to produce products that are toxic in high concentrations (e.g., biofuels), operate in stressful environments (e.g., high salt, low pH), or perform bioremediation. Here, Raman spectroscopy was further developed as a microbial phenotyping methodology, with the focus on analyzing responses of cyanobacteria to salt (NaCl and MgSO_4_) and acid (acetate) stressors.

In this context, the cyanobacterium *Synechocystis* sp. PCC6803 (hereafter *Synechocystis*) is of interest to biotechnology because of its ability to grow on low-cost resources (i.e., CO_2_ and sunlight) and produce high-value chemicals (Yu et al., 2013). This species demonstrates a highly versatile carbon metabolism, growing in autotrophic as well as under heterotrophic and mixotrophic conditions (Vermaas, 1996). It is also a model organism for many cyanobacteria, which are, in general, known to tolerate high salt concentrations (Reed et al., 1985). Acclimation of *Synechocystis* to high salt concentrations begins with adjustment of osmotic equilibrium with the outside medium (Marin et al., 2004; Marin et al., 2006; Karandashova & Elanskaya, 2005), followed by the accumulation of the osmoprotectant glycosylglycerol (Reed et al., 1986; Marin et al., 2004; Marin et al., 2006; Hagemann, 2013). This osmoprotectant enhances the internal osmotic potential and protects proteins and membranes (Borges et al., 2002; Hincha & Hagemann, 2004). In addition, it has been shown that long-term salt stress induces accumulation of specific proteins (Hagemann, Techel & Rensing, 1991; Hagemann & Erdmann, 1994; Fulda et al., 2000; Huang et al., 2006) and alterations in fatty acids composition (Huflejt et al., 1990; Khomutov et al., 1990; Allakhverdiev et al., 1999; Singh, Sinha & Häder, 2002).

*Synechocystis* has the ability to grow mixotrophically in the presence of glucose and acetate (Wu, Shen & Wu, 2002); however, utilization of acetate as the sole carbon source is limited as it lacks major enzymes required for uptake (Knoop et al., 2013; Varman et al., 2013; Thiel et al., 2017). Acetate could serve as a supplementary precursor for the synthesis of certain biomass building blocks such as amino acids (Varman et al., 2013; Thiel et al., 2017) and lipids (Thiel et al., 2017). Mechanisms of acetate toxicity have been studied mainly in Gram-negative bacteria and include alterations in cell membranes, active secretion, and activation of stress responses (Isken & De Bont, 1998; Sardessai & Bhosle, 2002). Magnesium, on the other hand, is an important cofactor for activities of many enzymes, while sulfur is a major macronutrient (0.6% of biomass dry weight) (Schwarz & Forchhammer, 2005; Kim et al., 2011). It is incorporated in proteins, lipids, and other vital compounds, and it is needed for the thioredoxin-mediated regulation of enzymes involved in photosynthesis and respiratory activities (Zhang et al., 2008; Kiyota, Ikeuchi & Hirai, 2012).

Commonly, biological studies of physiological responses to chemical stressors have taken place using genomic methods (Fulda et al., 2000; Marin et al., 2004; Karandashova & Elanskaya, 2005; Huang et al., 2006; Zhang et al., 2008) to study gene expression and biochemical approaches (Marin et al., 2006; Zavřel, Očenášová & Červený, 2017) to characterize biomolecules. These require extensive sample preparation and long analysis times. Near real-time methods of analysis are needed alongside standardized procedures to provide rapid information to further understand microbial metabolic responses to environmental stress or for phenotype screening purposes. With such methods, cultures could be monitored in near real-time in biotechnological applications so that the appearance of unhealthy or unproductive phenotypes could be remedied quickly.

Raman spectroscopy has been proven a powerful analytical technique for the analysis of biological materials. It uses monochromatic light to provide a qualitative measurement of the biochemical composition of the biological specimen (Butler et al., 2016) and gives distinctive signals from macromolecules (i.e., proteins, lipids, carbohydrates, and nucleic acids) (Das & Agrawal, 2011). Therefore, a characteristic fingerprint is assigned to each biological sample and allows a better understanding of its chemical composition. Raman spectroscopy allows near real-time and noninvasive acquisition of phenotype data from living systems (Das & Agrawal, 2011; Zu et al., 2014; Butler et al., 2016; Freedman et al., 2016; Zu, Athamneh & Senger, 2016; García-Timermans et al., 2019). In addition, the analysis is label free, requires minimal or no sample preparation, and there is no spectral interference from water (Zu et al., 2014; Freedman et al., 2016; Zu, Athamneh & Senger, 2016). The application of Raman spectroscopy is an expanding field and it includes fermentation monitoring (Sivakesava, Irudayaraj & Demirci, 2001; Ewanick et al., 2013; Olson et al., 2016; Zu et al., 2016), detection and identification of microorganisms (Nelson, Manoharan & Sperry, 1992; Kirschner et al., 2001; Pahlow et al., 2015), monitoring the kinetics of germination of individual *Clostridium difficile* spores (Wang et al., 2015) and detection of its toxins (Koya et al., 2018). It has been also demonstrated that Raman spectroscopy can be used in near-real time phenotyping of *Escherichia coli* exposed to alcohol (Zu et al., 2014; Zu, Athamneh & Senger, 2016) and antibiotics (Athamneh et al., 2014), single cell phenotyping (Wu et al., 2011; Serrano et al., 2014; Sun et al., 2015; García-Timermans et al., 2019), and characterizing phenotypic differences among *E. coli* enriched for 1-butanol tolerance (Freedman et al., 2016; Zu et al., 2014). Thus, Raman spectroscopy was selected as an effective method for probing changes in *Synechocystis* phenotypes in near real-time when exposed to external stimuli.

In this research, Raman spectroscopy was used to perform microbial phenotyping of *Synechocystis* cultures growing in different concentrations of (i) acetic acid (7.5–30 mM), (ii) NaCl (50–150 mM), and (iii) MgSO_4_ (0–62.5 mM) in BG-11 media. This was done to test the hypothesis that Raman spectroscopy and spectral processing with Raman chemometrics (Rametrix™) and statistical analyses could distinguish among phenotypes and quantify changes in biomolecular composition. Routine analytical methodology was used to measure the levels of several biomolecules (e.g., fatty acids and amino acids) so correlations could be made with individual Raman band intensities. This technique was shown previously (Zu et al., 2014) to correlate Raman data with fatty acid and amino acid analytical data for *Escherichia coli*. Here, this approach was expanded to include *Synechocystis* and the stress-induced phenotypes studied. *Synechocystis* was chosen for this study because it is a well-studied model organism for many cyanobacteria. It has well-developed genomic tools, relevance to biotechnology, and stress-response and chemical composition data available in the literature.

## Material and Methods

### Bacterial strain and culture conditions

*Synechocystis* sp. PCC6803 (ATCC^®^27184™) were used to create axenic kanamycin resistant mutants for the comparative growth experiments (Heidorn et al., 2011; Tanniche, Collakova & Senger, 2019, unpublished data). This strain was used with kanamycin (15 µg/mL) to help prevent contamination. Cells were cultivated in the BG-11 medium (Rippka et al., 1978) (adjusted to pH 7.0) supplemented with 5 mM glucose. Three different stress conditions were analyzed: (i) NaCl (50, 100, and 150 mM), (ii) acetate (7.5, 15, and 30 mM) and (iii) MgSO_4_ (0, 15.6, 31.2, and 62.5 mM; representing 0%, 25%, 50% and 100% of BG-11). Cultures grown in BG-11 were used as controls, and cells harvested in mid-exponential phase were used to initiate stress experiments. Each culture type condition experiment was performed in biological triplicate. Cultures were grown in 50 mL volume in 125 mL flasks, incubated at 25 °C under continuous light (20 µE), agitated at 140 rpm, and ambient CO_2_. Cells were harvested at mid-exponential phase and washed twice with ice-cold purified water. 2 µL of cells were kept for Raman scans and the remaining pellet was freeze-dried and prepared for analyses by UPLC and GC-FID.

### Raman spectroscopy

To prepare samples for Raman analysis, 2 µL of washed cells were dried at room temperature on aluminum foil (three samples per biological replicate). Dried cells were analyzed using a PeakSeeker PRO-785 Raman microscope (Agiltron; Woburn, MA) with 10X objective. Measurements were carried out using the following settings: laser excitation of 785 nm (30 mW) for 5 seconds with spectral resolution of 13 cm^−1^. Twenty individual scans were taken per sample by focusing on different areas of the dried cells. Raman data were collected using RSIQ™  software.

### Computational methods

Acquired Raman spectra were processed and analyzed using the Rametrix™ LITE Toolbox (Fisher et al., 2018) and Rametrix™ PRO Toolbox (Senger & Robertson, 2020) for MATLAB^®^. MATLAB^®^ R1018A with the Statistics and Machine Learning Toolbox was used for all calculations. Data analysis with Rametrix™ LITE Toolbox (Fisher et al., 2018) consisted of averaging the 20 spectra replicates and then baselining with the Goldindec algorithm (Liu et al., 2015) (baseline polynomial order = 3, estimated peak ratio = 0.5, and smoothing window size = 5) and vector normalizing over the biological range (400 cm^−1^–1,800 cm^−1^). PCA was performed, and outlier spectra were identified and excluded from analysis. Next, DAPC was applied, and several DAPC models were constructed by varying the number of principal components (PCs) used to build each one. DAPC models enabled the separation of Raman spectra according to a preset experimental factor. Here, the culture medium composition and environmental stimuli (acetate, NaCl, and MgSO_4_) concentrations were used. The Rametrix™ PRO Toolbox (Senger & Robertson, 2020) was used to apply leave-one-out analysis to DAPC clustering. This analysis determines the ability of DAPC models to predict correctly the classification when presented with an unknown Raman spectrum. The performance of DAPC models are quantitated by prediction accuracy (percent of unknown spectra classified correctly), sensitivity (true positive percentage), and specificity (true negative percentage).

Analysis of variance (ANOVA), pairwise comparisons using Tukey’s honest significant difference (HSD), and regression statistical analyses required the representation of the Rametrix™  LITE outputs (spectra intensity values, PCA, and DAPC) as a single numerical value. For example, the hundreds of intensity values at each Raman shift (400–1,800 cm^−1^) had to be reduced to a single numerical value for each spectrum. This is also true as data-rich spectra were reduced from hundreds of intensity values to tens of PCs by PCA and canonical values by DAPC. To calculate this single numerical value for statistical comparisons between spectra, the distance formula was applied to determine the similarity between two spectra, where one was a reference. When using PCA data, the Total Principal Component Distance (TPD) was calculated according to Eq. (1), where the first five PCs (representing more than 95% of the dataset variance) were used.

(1) \begin{eqnarray*}TPD=\sum _{i=1}^{5}\sqrt{{ \left( {P}_{x,i}-{P}_{reference,i} \right) }^{2}}.\end{eqnarray*}

Here, *P*_*x*,*i*_ is the *i*th PC of spectrum *x* and *P*_*reference*,*i*_ represents the *i*th PC of the reference. The calculation was repeated for all *x* spectra in the analysis. Different spectra were chosen as the reference in each of the four sub-studies presented in this research. These are identified for each in Appendix S1 in the Raw Distances and Mean Distances sheets. More details about TPD and its value in statistical calculations have been published (Senger et al., 2019). Similarly, the distance calculation was applied to each spectrum to calculate the Total Spectral Distance (TSD), based on normalized intensity values across all Raman shifts, and the Total Canonical Distance (TCD), based on the first five canonicals in the DAPC model. Reduction of spectral data into a single value allowed statistical analysis including analysis of variance (ANOVA), pairwise comparisons using Tukey’s honest significant difference (HSD), and regression. These analyses were performed to assess whether significant differences exist among the separated groups of spectra (ANOVA), to determine which group differences are statistically significant (pairwise comparisons), and establish a linear fit among data.

### Biomass measurements

Fatty acid content was determined by gas chromatography-flame ionization detection (GC-FID) after direct hydrolysis and conversion to fatty acid methyl esters (FAME). Lyophilized cells were incubated with methanolic HCl and 10 µg heptadecanoic acid (C17:0) as internal standard at 75 °C for 2 h to form FAME. GC-FID was done on an Agilent 7890A series GC equipped with FID. For fatty acid identification purposes, the same GC was coupled to a 5,975C series single quadrupole mass spectrometer (MS) and fatty acids were identified by comparing the spectra with the corresponding spectra in the NIST library (Agilent Technologies, Santa Clara, CA).

Protein hydrolysis was carried out in a custom-made teflon hydrolysis chamber (see photo in Appendix S2) under vacuum at 110 °C in the presence of 6N HCl. 50 mM norvaline was used as an internal standard. Amino acid levels were determined after derivatization with AccQ-Tag^TM^ reagent using a Waters Acquity^TM^H-class ultra-performance liquid chromatography (UPLC) system equipped with a fluorescent detector (Waters Corporation, Mildford, MA).

### Public access

The Rametrix™ LITE Toolbox is shared through GitHub with license agreement (https://github.com/SengerLab/RametrixLITEToolbox), and the Rametrix™ PRO Toolbox is also accessible through GitHub with license agreement (https://github.com/SengerLab/RametrixPROToolbox). Raw Raman spectra (in *.SPC file format) and biological measurement raw data are shared in the Supplemental Information 3.

## Results

### Raman spectroscopy of *Synechocystis* grown in the presence of acetate and salts

Raman spectroscopy was used to identify phenotypic changes in *Synechocystis* induced by the presence of: (i) acetate, (ii) NaCl, and (iii) MgSO_4_. Cells were scanned with Raman microscopy and the resulting spectra were analyzed using the Rametrix™  LITE (Fisher et al., 2018) and PRO (Senger & Robertson, 2020) Toolboxes. The obtained spectra were averaged, truncated (400–1,800 cm^−1^), baselined, and vector normalized prior to further processing using PCA and DAPC in the Rametrix™  LITE. Rametrix™  PRO was used to determine the ability to predict a particular metabolic phenotype given a Raman spectrum of cells grown under unknown growth conditions. Next, statistical analyses (i.e., ANOVA and pairwise comparisons) were performed on TCD and TPD results. TPD and TSD produced similar results, allowing TSD to be omitted in the analysis. Finally, specific Raman bands assigned to certain metabolites (i.e., amino acids and fatty acids) were compared to standardized analytical methods (i.e., UPLC, GC-FID). The remainder of the results section is divided according to analysis type: (i) Rametrix™  analyses and (ii) correlation of individual Raman bands with well-established analytical approaches.

### Rametrix™ analyses

#### Acetate-induced phenotypes

**Figure 1:**
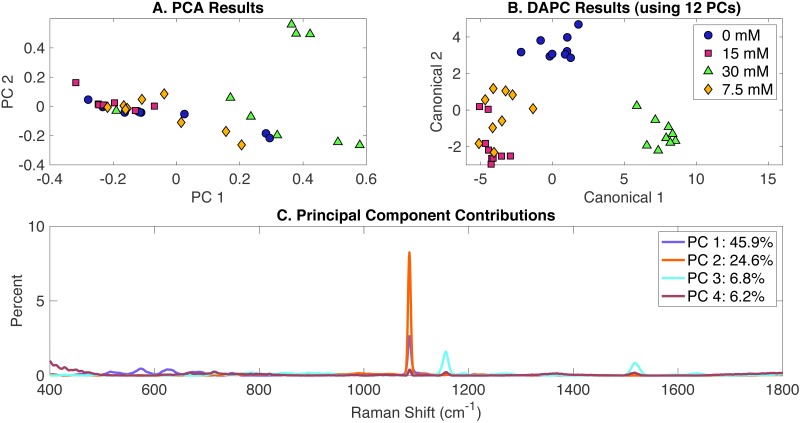
Rametrix™ LITE analysis of acetate-induced phenotypes. (A) PCA, (B) DAPC, and (C) Raman shifts contributions between groups in PCA. 0 mM represents native BG-11 medium.

Raman spectra of cultures growing in three concentrations of acetate (7.5 mM, 15 mM and 30 mM) and the control (BG-11 without acetate) were analyzed by Raman spectroscopy and the Rametrix™  LITE and Rametrix™  PRO Toolboxes. PCA, DAPC, and PC contributions results are shown in Fig. 1. PCA of Raman spectra (Fig. 1A) showed some separation of clusters in the two first PCs, which contained more than 68% of the dataset variance. This clustering is more noticeable with the DAPC model (Fig. 1B) constructed with 12 PCs (representing more than 99% of the dataset variance). Although four marked clusters were revealed, based on the concentration of acetate in the BG-11 medium, only three major groups were observed. These clusters suggest that phenotypic differences could be generated as a result of acetate addition to BG-11 medium and that cells grown in the presence of 7.5 mM and 15 mM could have similar phenotypic responses. Next, Raman shift contributions for both PCA and DAPC were studied to point to molecular differences between clusters of Raman spectra. In this particular case, it provided information about the molecular differences between cultures grown in the presence and absence of acetate. It does not provide information about accumulation or depletion of individual biomolecules (e.g., carotenoids), but it does suggest the associated band intensities change significantly. Raman shift contributions between groups in PCA are shown in Fig. 1C and the contributions associated with DAPC model are represented in Fig. S1. Raman band assignments were selected based on published libraries (De Gelder et al., 2007; Movasaghi, Rehman & Rehman, 2007; Zhu et al., 2011). The full list of assignments is given in Appendix S1, and highlights are given in Table 1.

**Table 1:** Highlighted Raman shift contributions to PCA and DAPC models.

Study	Model	Biomolecules and Raman bands (cm^−1^)
Acetate-induced	PCA	Glycerol (630 cm^−1^), amino acid (573 cm^−1^) protein (1,156 cm^−1^), and carotenoids (1,156, 1,518 cm^−1^)
Acetate-induced	DAPC	Proteins (524, 618, 1,005, 1,155 cm^−1^), carotenoids (1,155, 1,528 cm^−1^), amino acids (532, 638, 715, 920, 988, 1,005, 1,196 1,327, 1,495, 1,550, 1,558, 1,615 cm^−1^), phosphatidylinositol (415 cm^−1^), polysaccharides (477 cm^−1^), DNA/RNA (746, 1,510 cm^−1^), and lipids (1,086 cm^−1^)
NaCl-induced	PCA	Glycerol (630 cm^−1^); proteins, amides and lipids (1,260 cm^−1^); carotenoids (1,157, 1,520 cm^−1^); and amino acids (515, 573, 669, 684, 1,188 cm^−1^)
NaCl-induced	DAPC	Sterols (430 cm^−1^), phosphatidylinositol (594 cm^−1^), DNA/RNA (746 cm^−1^), DNA (1,090, 1,329 cm^−1^), amino acids (730, 854, 1,040, 1,495 cm^−1^), carotenoids (1,157, 1,517 cm^−1^), nucleic acids (650, 1,200, 1,329 cm^−1^), and lipids (1,255 cm^−1^)
MgSO_4_-induced	PCA	Sterols (429 cm^−1^), glycerol (630 cm^−1^), carotenoids and carotene (1,157, 1,520 cm^−1^), amino acids (573, 684, 1,188 cm^−^1), lipids (1,380 cm^−1^), and phospholipids (1,330 cm^−1^)
MgSO_4_-induced	DAPC	Carotenoids (1,157, 1,520 cm^−1^) and amino acids (850, 992 cm^−1^) in addition to phosphatidylinositol (412 cm^−1^) and saccharides (850, 868, 1,456 cm^−1^)

Next, TCD, TPD and TSD (both raw and mean values) were calculated using PCA and DAPC data and used in ANOVA and pairwise comparisons. Full results are given in the Appendix S1. In general, as the phenotype deviates from the reference (represented here by phenotypes of cells grown on BG-11 medium), the TCD (or the TPD) mean value should become larger. Similar phenotypes should have comparable values. In the acetate-induced phenotypes, TCD and TPD mean values increased proportionally to the increase of acetate concentration. Based on TCD data (using 12 PCs in DAPC model) ANOVA results indicated that differences between the groups are significant (*p* < 0.001). With TPD data, ANOVA returned a significant *p*-value (*p* < 0.001). This result indicates that even though no clear clustering was observed in PCA (Fig. 1A), there are statistically significant differences between the groups. Pairwise comparisons identified all phenotypes (groups) to be significantly different based on the TCD data (*p* < 0.001). However, using TPD data, 7.5 mM and 15 mM induced phenotypes were found not significantly different (*p* = 0.997). The small difference in mean TPD and the near clustering observed in Fig. 1B are consistent with this result. Next, regression (Appendix S1) was computed between acetate concentrations (0 mM, 7.5 mM, 15 mM, 30 mM) and the TCD and TPD mean values, respectively. Results show a linear relationship among the data with a coefficient of determination (*R*^2^) of 0.97 and 0.84 for TPD and TCD, respectively.

Rametrix™ PRO was used to apply leave-one-out analysis to the DAPC clustering in Fig. 1B. The purpose of this analysis is to determine the ability of the DAPC model to predict precisely the classification (i.e., 0 mM, 7.5 mM, 15 mM, 30 mM of acetate) when presented with a Raman spectrum of *Synechocystis* cells grown in an unknown condition. Rametrix™ PRO results are also given in Appendix S1, and more details of Rametrix™ PRO calculations are given in Senger & Robertson (2020). Here, four classification options, 0 mM, 7.5 mM, 15 mM and 30 mM, were assessed. With four different classifications, the random chance specificity was calculated as 25% and the random chance specificity as 75% for this dataset. Therefore, model performance sensitivity and specificity must be higher than these values if the model is truly able to predict *Synechocystis* phenotypes based on Raman spectra. Highlighted Rametrix™ PRO results are given in Table 2, and all sensitivity and specificity values exceeded random chance values, with specificity and sensitivity values ranging between 56–100% and 81–100%, respectively.

**Table 2:** Highlighted Rametrix™ PRO results.

Study	Classification predicted	Sensitivity	Specificity
All Studies	Random chance^**^	25%	75%
Acetate-induced	0 mM	100%	89%
Acetate-induced	7.5 mM	67%	81%
Acetate-induced	15 mM	56%	100%
Acetate-induced	30 mM	56%	96%
NaCl-induced	0 mM	100%	100%
NaCl-induced	50 mM	33%	96%
NaCl-induced	100 mM	44%	100%
NaCl-induced	150 mM	44%	89%
MgSO_4_-induced	62.5 mM	100%	100%
MgSO_4_-induced	31.2 mM	89%	96%
MgSO_4_-induced	15.6 mM	100%	89%
MgSO_4_-induced	0 mM	56%	78%
All phenotypes	BG-11 control	100%	100%
All phenotypes	Acetate-induced	56%	100%
All phenotypes	NaCl-induced	71%	95%
All phenotypes	MgSO_4_-induced	93%	100%

**Notes.**

* All DAPC models were built with 12 PCs representing 99% of the dataset variance.

** The random chance sensitivity and specificity values were calculated statistically, not by RametrixTM PRO. They were the same for all studies here.

### NaCl-induced metabolic phenotypes

**Figure 2:**
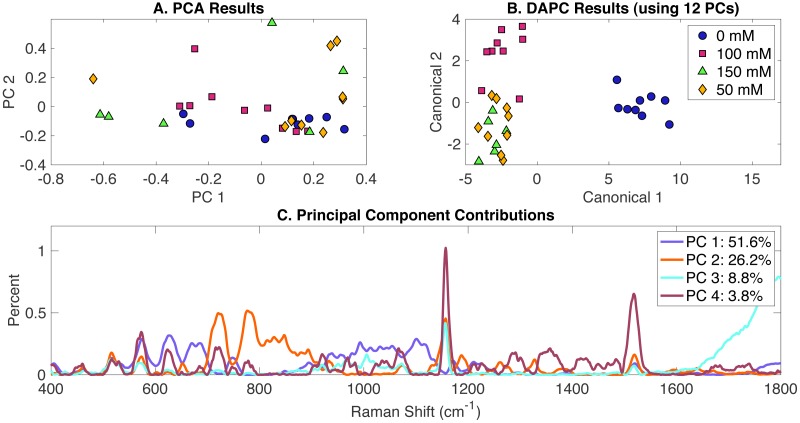
Rametrix™ LITE analysis of NaCl-induced phenotypes. Rametrix™ LITE analysis of NaCl-induced phenotypes. (A) PCA, (B) DAPC, and (C) Raman shifts contributions between groups in PCA. 0 mM represents native BG-11 medium.

In order to determine the effect of NaCl addition to BG-11 medium on *Synechocystis* metabolic phenotypes, cells were grown in BG-11 at three salt concentrations (50 mM, 100 mM, and 150 mM) and compared to a control (cells grown in BG-11 alone with no NaCl added). PCA, DAPC and PC contribution results generated by the Rametrix™ LITE are represented in Fig. 2. PCA of Raman spectra (Fig. 2A), again, showed no apparent clustering in the first two PCs, which comprised about 69% of the dataset variance. However, the application of DAPC (Fig. 2B) demonstrated four clusters representative of the three different concentrations of NaCl and the control. These results were obtained using 12 PCs (representing over 99% of the dataset variance). Based on the presence of NaCl, two major groups were noticed (Fig. 2B), suggesting significant cellular phenotype differences could occur in response to as little as 50 mM NaCl in BG-11 medium. Next, Raman shift contributions to the PCA and DAPC results were examined. Raman shift contributions between the groups in PCA are shown in Fig. 2C, and those related to DAPC contributions are represented in Fig. S2. Specific lists of Raman band contributions and molecular assignments (for both PCA and DAPC) are given in Appendix S1, and highlights are given in Table 1.

DAPC model and PCA were used to compute TCD, TPD, TSD, and their mean values. Multi-way ANOVA and pairwise comparisons were used to assess these parameters, and full results are given in Appendix S1. As mentioned previously, the TCD and TPD mean values increase with the increased difference from the reference phenotype. Using the TPD mean values, as the concentration of NaCl increased, the distance also increased. However, a small difference was observed between the 50 mM and the 100 mM TPD means, suggesting comparable phenotypes. The TCD mean values and the clustering in the Fig. 2B confirm this result. ANOVA revealed that molecular phenotypes induced at different NaCl concentrations were significantly different (*p* < 0.001) for TCD and TPD data. Pairwise comparison allowed the identification of significantly different phenotypes. Based on the TPD data, the phenotypic responses associated with 100 mM NaCl were not significantly different from the BG-11 medium (*p* = 0.165) and 50 mM (*p* = 0.091). Pairwise comparisons also showed that the phenotypes observed at 150 mM NaCl were not statistically different from the phenotypes at 50 mM (*p* = 0.04). On the other hand, based on the TCD data, the phenotypes resulting from 50 mM, 100 mM, and 150 mM NaCl treatments were all statistically different from the BG-11 medium control (all *p*-values < 0.001), but they were not different from one another (all *p*-values >0.027). Finally, Regression (Appendix S1) was applied between NaCl concentrations and the TCD and TPD mean values. A coefficient of determination (*R*^2^) of 0.64 and 0.51 were obtained for TPD and TCD, respectively.

Rametrix™ PRO was used to apply leave-one-out analysis to DAPC in Fig. 2B. Similar to the previous analysis, four classification options were included: 0 mM, 50 mM, 100 mM, and 150 mM NaCl. Each of these conditions was selected as the positive condition to generate accuracy, sensitivity, and specificity results. As mentioned previously, sensitivity and specificity values should exceed those of the random chance values to confirm the model has value in identifying *Synechocystis* phenotypes. Highlighted results for a DAPC model built with 12 PCs (representing more than 99% of dataset variability) are given in Table 2. Results for additional DAPC models are given in Appendix S1. The best-performing model (Table 2) returned sensitivity and specificity values that exceeded the random chance values. In addition, 0 mM classification showed the highest sensitivity and specificity rates (100%). This suggests that NaCl-induced phenotypes may occur below 50 mM and remain somewhat similar through 150 mM. The BG-11 control phenotypes were significantly distinguishable from the ones observed for the other three conditions, as shown in Fig. 2B.

### MgSO_4_-induced phenotypes

**Figure 3:**
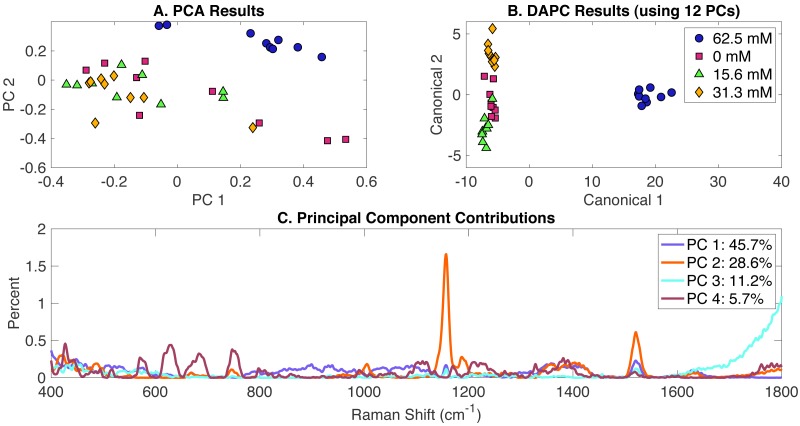
Rametrix™ LITE analysis of MgSO_4_-induced phenotypes. Rametrix™ LITE analysis of MgSO_4_-induced phenotypes. (A) PCA, (B) DAPC, and (C) Raman shifts contributions between groups in PCA. 62.5 mM represents native BG-11 medium.

*Synechocystis* cells growing under different levels of MgSO_4_ present in BG-11 medium were analyzed using Raman spectroscopy. PCA, DAPC and PCA contributions generated by the Rametrix™ LITE Toolbox are presented in Fig. 3. Again, no clustering was observed with PCA of Raman spectra (Fig. 3A) in the first two PCs (representing over 75% of the dataset variance). Application of DAPC using 12 PCs (representative of more than 99% of the dataset variance) revealed four distinguishable clusters (Fig. 3B), based on the concentration of MgSO_4_ in BG-11 medium. These clusters formed two separate groups representing cells grown under limiting concentrations of MgSO_4_ and BG-11 medium, confirming that limiting MgSO_4_ in the medium is linked with altering cellular phenotypes. Analysis of Raman shift contributions to the PCA and DAPC groups was also performed. Full results are given in Appendix S1, and highlights are given in Table 1. DAPC contributions are also identified in Fig. S3.

TSD, TCD and TPD values were calculated based on spectra, DAPC, and PCA results. These values were analyzed further by ANOVA and pairwise comparisons. All results are available in the Appendix S1. In general, TCD and TPD increased with the decrease of MgSO_4_ concentration, with the 15.6 mM classification mean values being closer to the reference when using the TPD means, compared to TCD mean values. Next, ANOVA and pairwise comparison tests were performed. It was found that differences between the groups (in both DAPC and PCA) are statistically significant (*p* < 0.001). Pairwise comparisons allowed the identification of significantly different phenotypes. With both TPD and TCD data, all phenotypes were different from those observed when cells were grown in the original BG-11 medium containing 62.5 mM MgSO_4_. These results confirm that even a 50% reduction in MgSO_4_ from the original BG-11 medium gives rise to various phenotypes. Results also show that phenotypes associated with the reduced MgSO_4_ content (31 mM) are not significantly different from 0 mM (*p* = 0.096) and phenotypes with even more reduced content (15.6 mM) (*p* = 0.032), when using TPD data. Similar results were found when using TCD values. Regression analysis (Appendix S1) between MgSO_4_ concentrations and both TCD and TPD mean values was performed. Results show linear relationships between the data for both cases with a correlation coefficient (R) of 0.81 and 0.76 for TPD and TCD, respectively.

For Rametrix™ PRO leave-one-out analysis on the DAPC model (Fig. 3B), four classification options were included for the four tested concentrations of MgSO_4_. Each one of these conditions was treated as a positive condition to generate accuracy, sensitivity, and specificity results. Again, results for several DAPC models are given in Appendix S1, and results for the DAPC model generated with 12 PCs (the best performer) are given in Table 2. Results confirmed the BG-11 cluster (62.5 mM MgSO_4_) (100% sensitivity and specificity) is significantly different from the other clusters. Again, all sensitivity and specificity values exceeded random chance, meaning the DAPC model was capable of applying these classifications for an unknown Raman spectrum of *Synechocystis* with accuracy greater than random chance.

### Classification of all phenotypes

**Figure 4:**
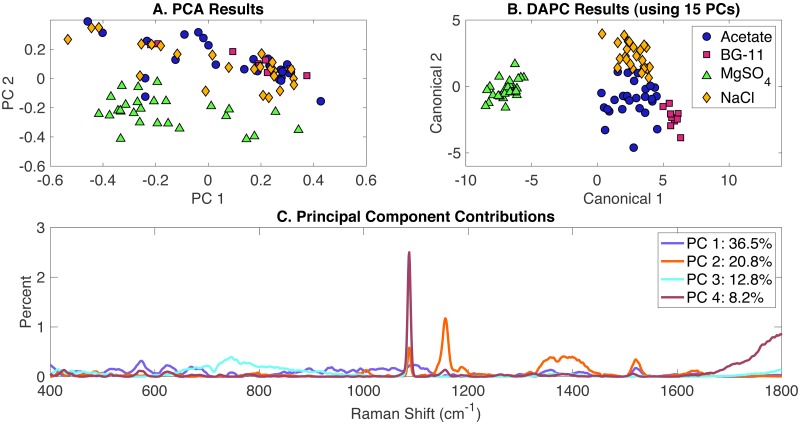
Rametrix™ LITE analysis of all phenotypes. Rametrix™ LITE analysis of all phenotypes. (A) PCA, (B) DAPC, and (C) Raman shifts contributions between groups in PCA.

All Raman scans performed in this study were compiled in a single dataset and included the growth conditions in the presence of the following environmental stimuli: (i) acetate, (ii) NaCl, (iii) MgSO_4_ and (iv) BG-11 medium (set as the control). Data analysis included the generation of PCA and DAPC models and validation with the leave-one-out analysis using Rametrix™ LITE and Rametrix™ PRO Toolboxes, respectively. PCA results are given in Fig. 4A and show some clustering, based on the two first two PCs which covered over 59% of the dataset variance. A DAPC model was constructed based on 15 PCs (representing over 99% of the dataset variance) and indicates four distinct clusters based on the presence or absence of environmental stimuli in the BG-11 medium (Fig. 4B). These clusters could be the result of potential differences in the cellular phenotypes obtained with every growth condition. Most importantly, a major segregation was observed for cells grown under MgSO_4_, clearly separating from the other conditions. Raman shift contributions to PCA (Fig. 4C) and DAPC groups (Fig. S4) are listed in the Appendix S1.

The analysis of TCD and TPD, using the BG-11 medium as a reference, allowed the classification of phenotypes. Both TCD and TPD mean values indicated the distance from the control (indicating more different phenotypes): (i) acetate-, (ii) NaCl-, and (iii) limited MgSO_4_-induced phenotypes. It appears that MgSO_4_ limitation generated the most significant changes in the cellular phenotypic responses (also shown in Fig. 4B). In addition, multi-way ANOVA indicated statistically significant differences between the groups representing each phenotype (*p* < 0.001). Using TPD data, pairwise comparisons indicated that all groups are significantly different from each other, only acetate (over all concentrations together) was not statistically different from BG-11 (*p* = 0.021). Based on TCD data (using 15 PCs), all phenotypes are significantly different (*p* < 0.001).

Next, Rametrix™ PRO was applied to determine if the phenotypes generated by the DAPC model could be predicted from Raman spectra (Appendix S1). Results are highlighted in Table 2. Again, all results exceeded the random chance values, indicating ability by the DAPC model to identify environmental stimuli exposure with accuracy much better than random chance.

### Correlation of individual Raman bands with well-established analytical approaches

Another method of comparing phenotypes is analysis of Raman bands assigned to functional groups of biomolecules. These signals are then correlated with results from accepted analytical methods such as GC-FID and UPLC for analyzing the levels of fatty acids and amino acids, respectively. The analysis of individual Raman band intensities was conducted based on the sets of Raman bands identified in previous research (De Gelder et al., 2007; Movasaghi, Rehman & Rehman, 2007; Zhu et al., 2011) and all band assignment are available in Appendix S2.

### Acetate study

The levels of amino acids (protein-derived and free amino acids) were determined by both Raman spectroscopy and UPLC analysis (Fig. S5). The general trend is an increase in amino acids levels for the three tested concentration of acetate (7.5 mM, 15 mM, 30 mM) compared to the control with an over-production of amino acids at 15 mM acetate and comparable levels at 7.5 mM and 30 mM acetate.

Raman assigned bands for the different amino acids (De Gelder et al., 2007; Zhu et al., 2011) were correlated with the UPLC data (Fig. S6). For most amino acids, correlation coefficients (R) varying between 0.61 and 0.97 were obtained. Ala, Leu, Lys, Met, and Ser had correlations with values of R ranging from 0.43 to 0.59. Since the acidic protein hydrolysis method used to release amino acid prevents from determining Cys and Trp levels (Olcott & Fraenkel-Conrat, 1947; Hugli & Moore, 1972; Yamada, Moriya & Tsugita, 1991), these amino acids were predicted using Raman band assignment alone (Fig. S7). The same trend was observed when Cys and Trp levels were higher at 15 mM of acetate.

Similarly, fatty acid content was evaluated and then compared to Raman bands (De Gelder et al., 2007; Czamara et al., 2015). Overall, there was an increase in fatty acid levels, mainly for C16:0, C18:2 and C18:3, where, at 15 mM acetate, levels of these fatty acids were the highest (Fig. S8). However, minor changes were observed for C16:1 and C18:1. Next, Raman signals corresponding to those fatty acids were compared with the GC-FID results (Fig. S9). Correlations were observed for C16:0 (*R* = 0.92) and C18:2 (*R* = 0.82), and correlation coefficients of 0.67 and 0.68 were found for C18:1 and C18:3, respectively. C16:1 had a correlation 0.46.

### NaCl study

Cells growing in different concentrations of NaCl were examined for their amino acid and fatty acid levels with UPLC and GC-FID, respectively. Raman signals corresponding to these biomolecules were used for comparison. Amino acid analysis revealed an increase in their levels proportionally to the amount of NaCl supplied to the media with a distinguishable over production of amino acids in the cells grown in the presence of 150 mM NaCl (Fig. S10). Comparison with the associated Raman bands showed correlation between the UPLC and Raman microscopy data (Fig. S11), where correlation (*R*) values were between 0.7–0.99, and slightly less correlation was observed for Val (*R* = 0.64). As stated previously, Cys and Trp are only predicted using Raman spectroscopy since they degrade during acidic hydrolysis (Olcott & Fraenkel-Conrat, 1947; Hugli & Moore, 1972; Yamada, Moriya & Tsugita, 1991). Again, based on the Raman assignments, we were able to resolve the patterns of these two amino acids, showing an increase in their levels mainly at 150 mM of NaCl (Fig. S12).

Finally, GC-FID analysis of the levels of free and lipid-derived fatty acids (Fig. S13) revealed an increase in the levels of C16:0, C18:2, and C18:3 in cells subjected to 50 mM of NaCl. In addition, no changes were observed for C16:1 and C18:1 levels. These results were compared with specific Raman bands assigned to each fatty acid. With the exception of C18:2, correlations (*R*) between 0.7 and 0.95 were obtained. A lower correlation was observed for the C18:2 between the Raman and GC-FID data (*R* = 0.28). This value improved to 0.7 when the 0 mM NaCl data point was removed from the analysis.

### MgSO_4_ study

The correlation of the individual Raman bands with their associated biomolecules could not be achieved for this study. MgSO_4_ is the major source of both elements in the BG-11 medium and sufficient amounts of the biomass could not be obtained for cells deprived of this salt, which prevented UPLC and GC-FID analyses.

## Discussion

Newly developed methods of analysis by Raman spectroscopy using Rametrix^TM^ enabled the assessment of phenotypic variations among *Synechocystis* cells growing in the presence of external substrates or stressors (acetate, NaCl, and MgSO_4_). Two different approaches of analysis were undertaken: (i) overall metabolic phenotype changes using Rametrix™ chemometric methods and (ii) correlation of specific Raman bands with their corresponding biomolecules and comparisons with data obtained by well-established analytical methods (i.e., UPLC and GC-FID). While more advanced instrumentation and single-cell methodologies exist (Wu et al., 2011; Serrano et al., 2014), the purpose of this research was to develop phenotyping capabilities with a relatively inexpensive and portable Raman microscope (with 10× objective imaging) that could have broad appeal to microbiology labs. Several Raman devices are now capable of field research, and Rametrix™ technologies may be able to be implemented there as well.

*Synechocystis* can grow in the presence of acetate in heterotrophic conditions (Wu, Shen & Wu, 2002). However, this microorganism cannot utilize acetate efficiently as it does not express necessary transporters (Knoop et al., 2013; Varman et al., 2013; Thiel et al., 2017). Phenotype changes due to different concentrations of acetate were observed through Rametrix™ LITE clustering of Raman spectral data and validated through statistical analyses (ANOVA and pairwise comparisons). Rametrix™ PRO displayed higher values of sensitivity and specificity (100% and 89%, respectively) over random chance values, supporting that our model can predict effectively the level of acetate in the culture media. Previous studies revealed that acetate is used for the biosynthesis of amino acids (Varman et al., 2013; Thiel et al., 2017) and lipids (Thiel et al., 2017) which is in agreement with our results (both with Raman spectroscopy and standard analytical methods). An over-accumulation of amino acids and fatty acids was observed mainly for acetate concentration of 15 mM. However, addition of 30 mM acetate into the BG-11 medium did not have a stronger effect compared to the 15 mM acetate. It is obvious that the amount of acetate supplied is limited by diffusion through the cell membrane (Knoop et al., 2013; Varman et al., 2013; Thiel et al., 2017) and that 15 mM might be the maximum concentration that could induce changes in the levels of the tested biomolecules. This result does not exclude the changes in the phenotypic responses observed with the clustering in Rametrix™ LITE and suggest that other changes might happen at 30 mM acetate. According to DAPC contributions, these changes include altered polysaccharide composition, and the levels and types of nucleotides and carotenoids.

NaCl-induced phenotypes were clearly different among the cells grown in the presence of different concentrations of this salt. ANOVA confirmed these results and pairwise comparisons revealed which phenotypes are significantly different from each other. In addition, Rametrix™ PRO was able to predict with accuracy, sensitivity (100%) and specificity (100%) greater than random chance the concentration of NaCl based on an unknown Raman spectrum of *Synechocystis* cells. Data from both Raman and the chromatography-based methods showed high correlations and were in an agreement with previously published data. In fact, salt stress triggers specific cellular responses, including synthesis of stress-related proteins (Hagemann, Techel & Rensing, 1991; Hagemann & Erdmann, 1994; Fulda et al., 2000; Huang et al., 2006) and alterations in fatty acid composition (Huflejt et al., 1990; Khomutov et al., 1990; Allakhverdiev et al., 1999; Singh, Sinha & Häder, 2002). In particular, 150 mM NaCl induces an over-production of amino acids (exceeding a 15-fold increase compared to the control) and significant changes in fatty acid composition.

Clustering of Raman spectral data via Rametrix™ LITE allowed the differentiation of metabolic phenotypes among *Synechocystis* cells in the presence of MgSO_4_-limiting concentrations. These results were validated by ANOVA and pairwise comparison. In addition, the DAPC model could predict the MgSO_4_ levels in the culture media with 100% specificity and sensitivity. The importance of Raman spectroscopy is well-demonstrated also as it provided valuable information with a limited biomass sample when the traditional analytical approaches could not be implemented. In addition, Raman shift contributions to the PCA and DAPC allowed the identification of specific biomolecules that contributed to the phenotypic changes observed with Rametrix™ LITE. These biomolecules included carotenoids, amino acids, lipids, phospholipids, and saccharides. Specific responses observed in photosynthetic organisms during acclimation to Mg and S deprivation include glycogen accumulation (Schwarz & Forchhammer, 2005), changes in amino acids composition (Kiyota, Ikeuchi & Hirai, 2012) as well as lipids (Sato et al., 2017) and degradation of photosynthetic components (i.e., chlorophyll a, PBS and carotenoids) (Jensen & Rachin, 1984; Richaud et al., 2001; Schmidt, Erdle & Köst, 2014). Interestingly, most of these biomolecules were identified with Raman shift contributions, suggesting that our findings are in an agreement with previously established results.

## Conclusions

Raman spectroscopy and analysis with the Rametrix™ Toolboxes and statistical tests enabled the identification of phenotypic changes in cells growing in the presence of different concentrations of external stressors or nutrients. In general, correlations were observed between Raman band intensities and measurements obtained with well-established analytical methods. Most importantly, Raman spectroscopy enabled the identification of the major biomolecules contributing to the phenotypic changes in samples with limited biomass, demonstrating its usefulness.

##  Supplemental Information

10.7717/peerj.8535/supp-1 Appendix S1 Raman band assignments: Rametrix™  and statistical calculations

10.7717/peerj.8535/supp-2 Appendix S2 Results of analytical experiments Figure S1–S4 are represented in Appendix 1.

10.7717/peerj.8535/supp-3 Supplemental Information 3 Raman scan files and analytical experiment measurements

## Additional Information and Declarations

### Data Availability

The following information was supplied regarding data availability: The Rametrix™ LITE Toolbox is available at GitHub under the MIT license (https://github.com/SengerLab/RametrixLITEToolbox), and the Rametrix™ PRO Toolbox is also accessible through GitHub under the MIT license (https://github.com/SengerLab/RametrixPROToolbox). Raw Raman scans and biological measurement raw data are available in the Supplementary File.
