# Supplementary material for: Characterizing metabolic stress-induced phenotypes of Synechocystis PCC6803 with Raman spectroscopy
Source: PeerJ. 2020 Mar 30;8:e8535. doi: 10.7717/peerj.8535 (PMC7115747; doi:10.7717/peerj.8535)
Supplement: Appendix S2 — Figure S1–S4 are represented in Appendix 1. [file peerj-08-8535-s002.pdf]

Supplementary Appendix 2

Characterizing metabolic stress-induced phenotypes of *Synechocystis* PCC6803 with Raman spectroscopy

Imen Tanniche[1], Eva Collakova[2], Cynthia Denbow[2], Ryan S. Senger*[1,3]

[1] Department of Biological Systems Engineering, Virginia Polytechnic Institute and State University (Virginia Tech), Blacksburg, Virginia, United States

[2] School of Plant & Environmental Sciences, Virginia Polytechnic Institute and State University (Virginia Tech), Blacksburg, Virginia, United States

[3] Department of Chemical Engineering, Virginia Polytechnic Institute and State University (Virginia Tech), Blacksburg, Virginia, United States

* Corresponding Author
1230 Washington St.
301C HABB1
Blacksburg, VA 24061
Email: senger@vt.edu
Phone: 540-231-9501

Note: **Figure S1-S4** are represented in Supplementary Appendix 1.

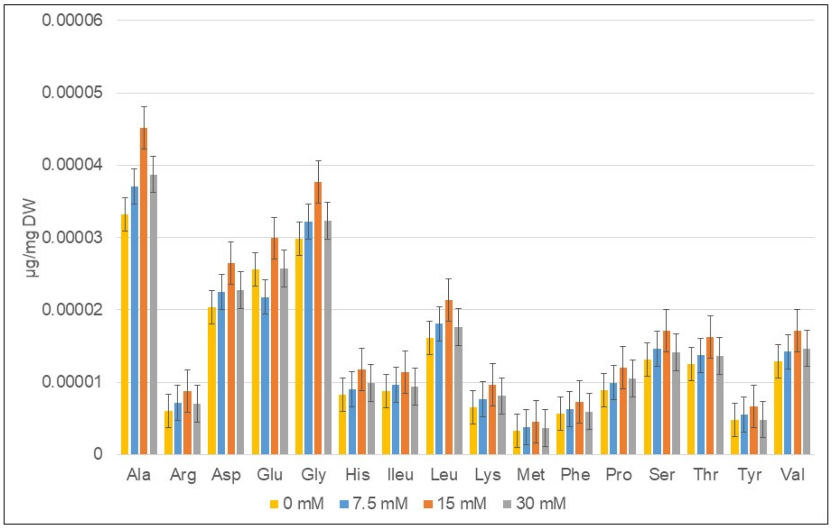

Figure S5. Amino acids analysis for acetate study. Data represent means ± standard deviation (SD) of values from three independent experiments.

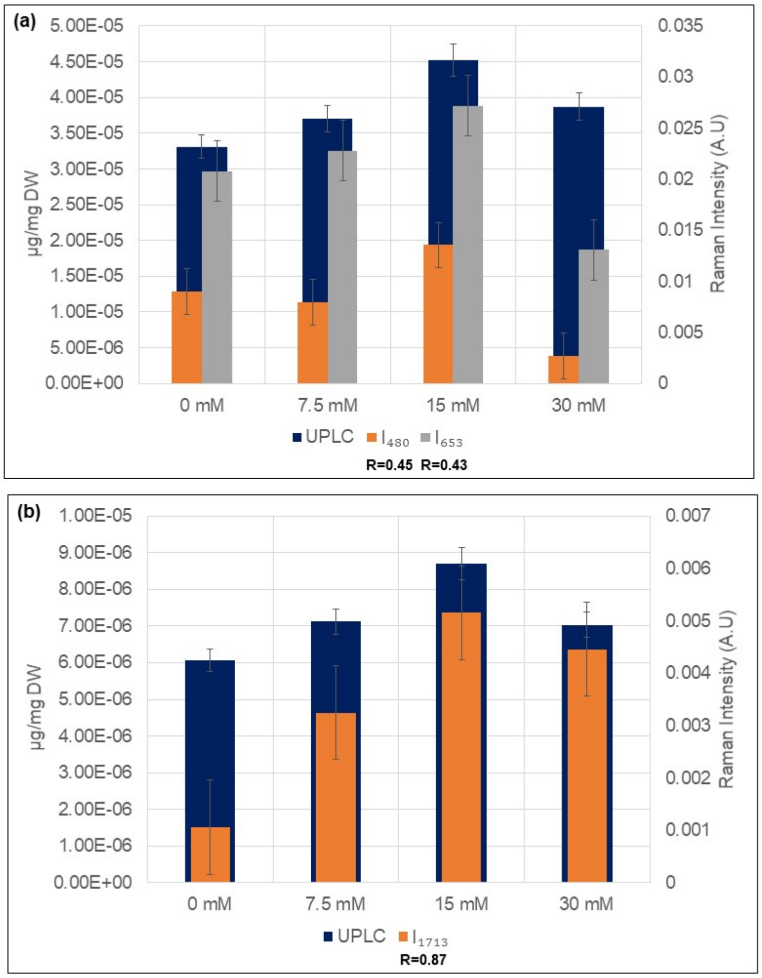

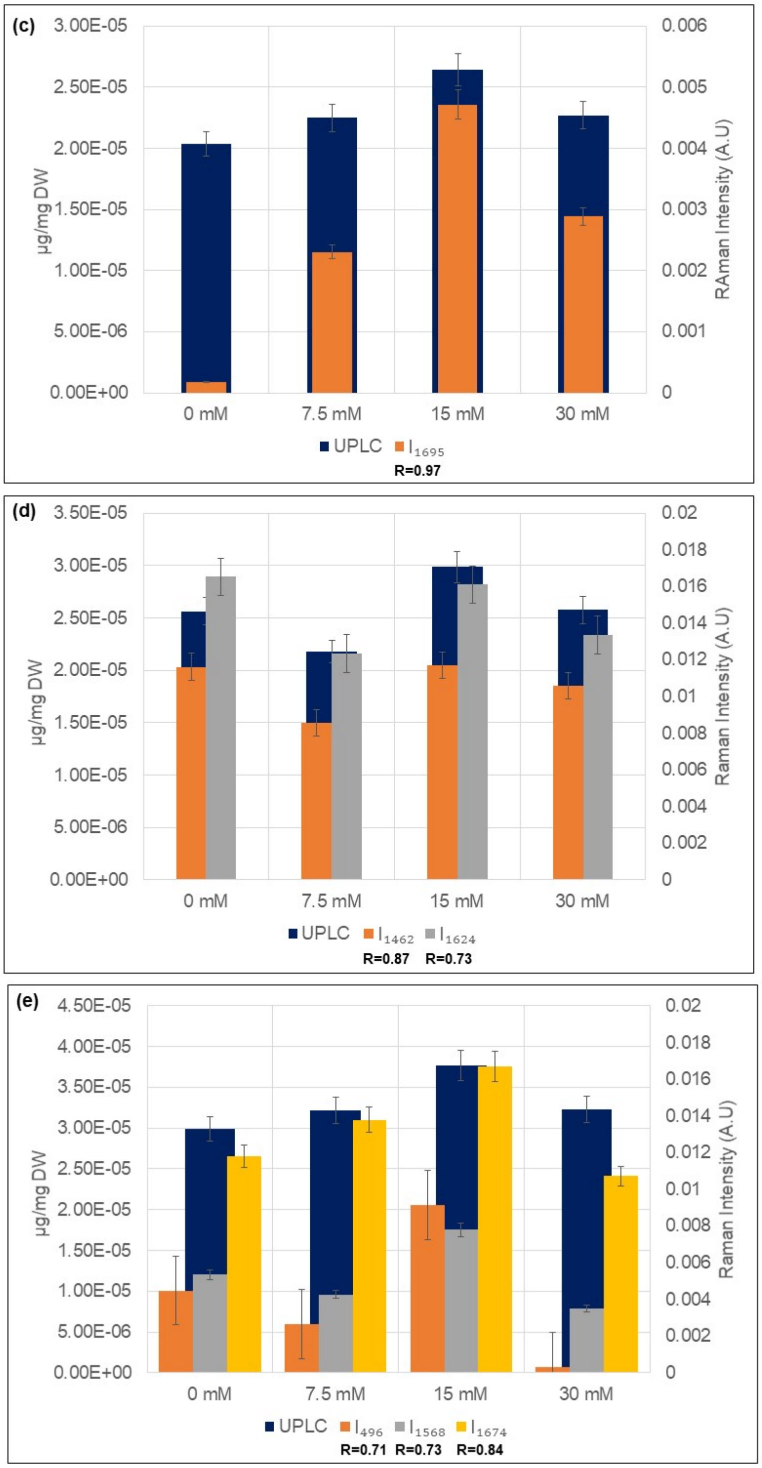
(c)
μg/mg DW
RAman Intensity (A.U)
0 mM
7.5 mM
15 mM
30 mM
UPLC
I1695
R=0.97
(d)
μg/mg DW
Raman Intensity (A.U)
0 mM
7.5 mM
15 mM
30 mM
UPLC
I1462
I1624
R=0.87
R=0.73
(e)
μg/mg DW
Raman Intensity (A.U)
0 mM
7.5 mM
15 mM
30 mM
UPLC
I496
I1568
I1674
R=0.71
R=0.73
R=0.84

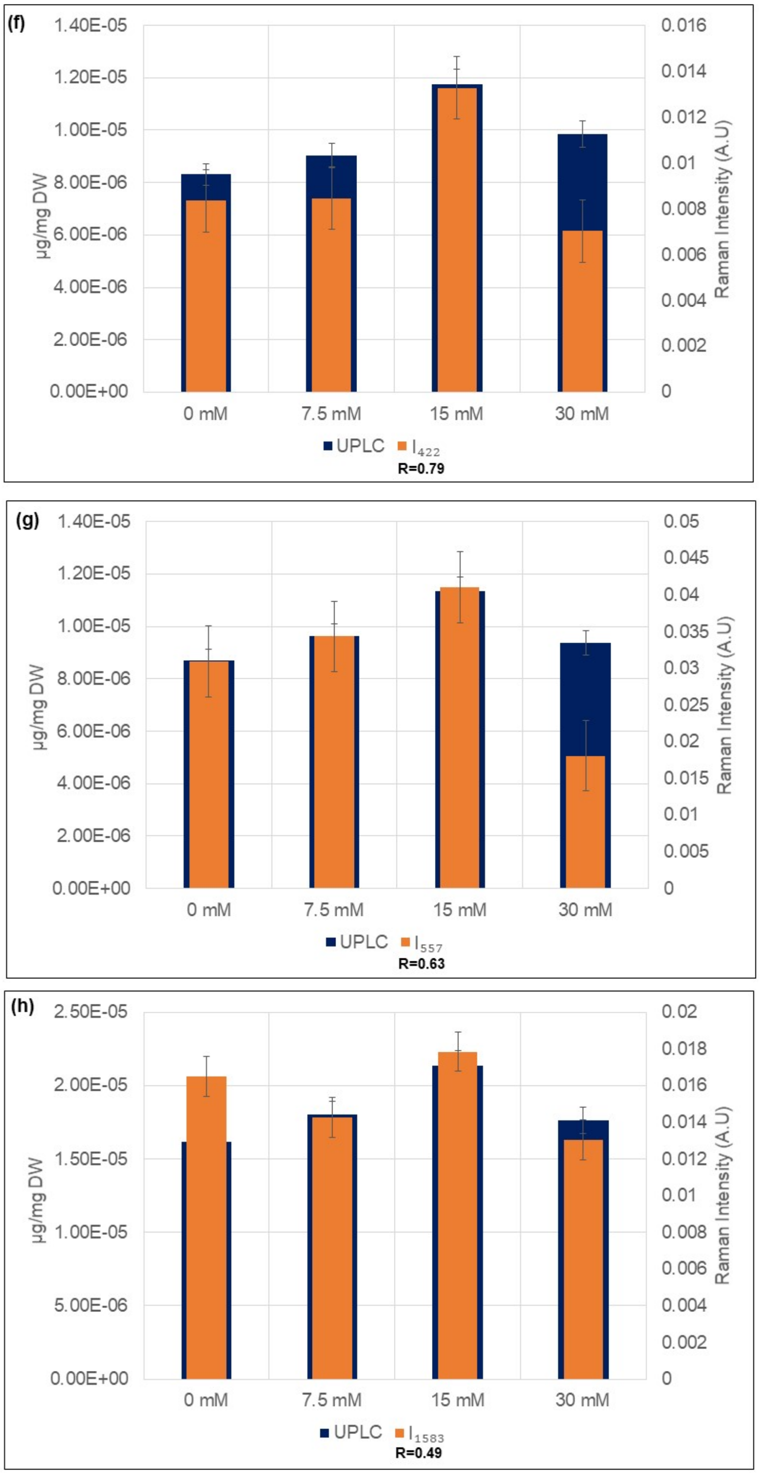
(f)
1.40E-05
1.20E-05
1.00E-05
8.00E-06
6.00E-06
4.00E-06
2.00E-06
0.00E+00
µg/mg DW
0.016
0.014
0.012
0.01
0.008
0.006
0.004
0.002
0
Raman Intensity (A.U)
0 mM
7.5 mM
15 mM
30 mM
UPLC
I_{422}
R=0.79
(g)
1.40E-05
1.20E-05
1.00E-05
8.00E-06
6.00E-06
4.00E-06
2.00E-06
0.00E+00
µg/mg DW
0.05
0.045
0.04
0.035
0.03
0.025
0.02
0.015
0.01
0.005
0
Raman Intensity (A.U)
0 mM
7.5 mM
15 mM
30 mM
UPLC
I_{557}
R=0.63
(h)
2.50E-05
2.00E-05
1.50E-05
1.00E-05
5.00E-06
0.00E+00
µg/mg DW
0.02
0.018
0.016
0.014
0.012
0.01
0.008
0.006
0.004
0.002
0
Raman Intensity (A.U)
0 mM
7.5 mM
15 mM
30 mM
UPLC
I_{1583}
R=0.49

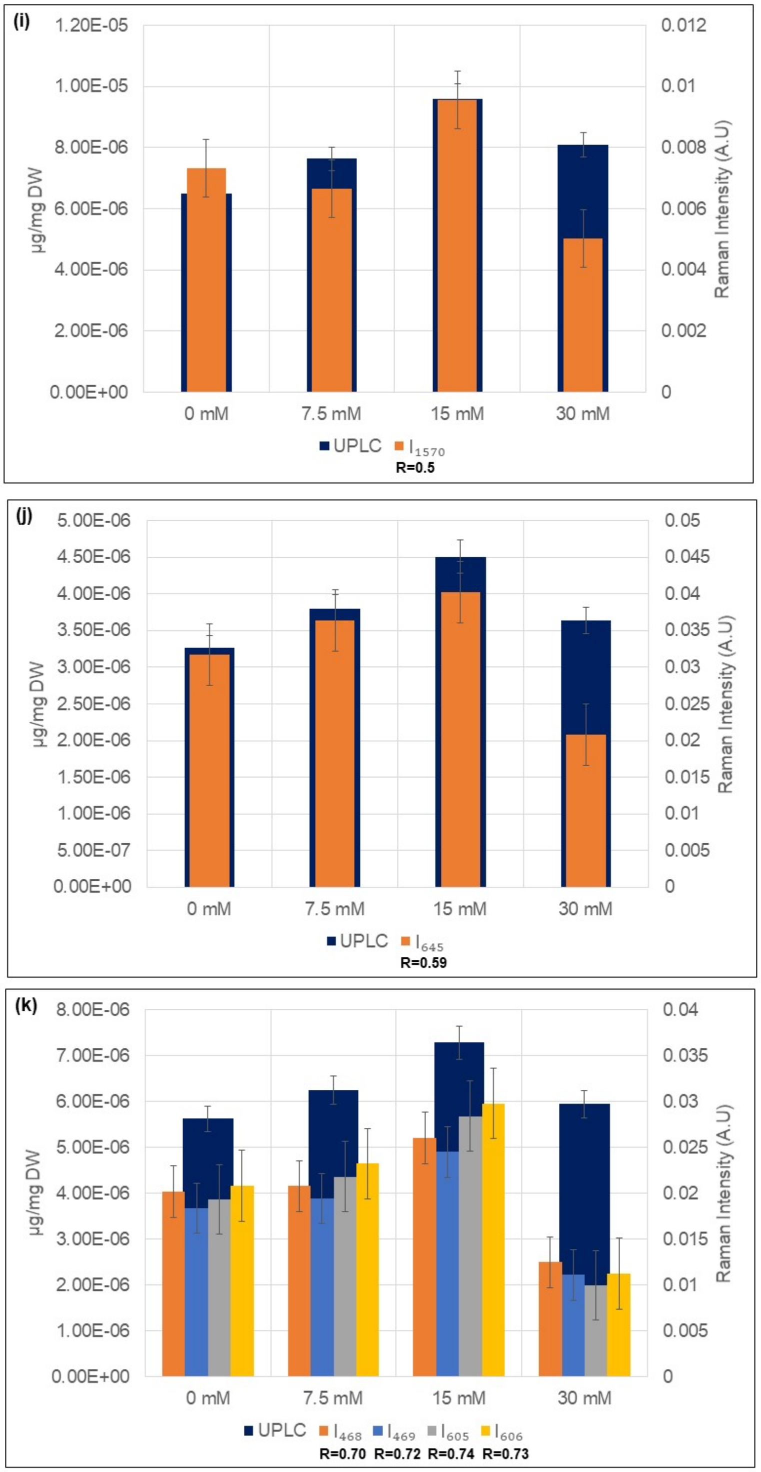
(i)
1.20E-05
1.00E-05
8.00E-06
6.00E-06
4.00E-06
2.00E-06
0.00E+00
µg/mg DW
0.012
0.01
0.008
0.006
0.004
0.002
0
Raman Intensity (A.U)
0 mM
7.5 mM
15 mM
30 mM
UPLC
I_{1570}
R=0.5
(j)
5.00E-06
4.50E-06
4.00E-06
3.50E-06
3.00E-06
2.50E-06
2.00E-06
1.50E-06
1.00E-06
5.00E-07
0.00E+00
µg/mg DW
0.05
0.045
0.04
0.035
0.03
0.025
0.02
0.015
0.01
0.005
0
Raman Intensity (A.U)
0 mM
7.5 mM
15 mM
30 mM
UPLC
I_{645}
R=0.59
(k)
8.00E-06
7.00E-06
6.00E-06
5.00E-06
4.00E-06
3.00E-06
2.00E-06
1.00E-06
0.00E+00
µg/mg DW
0.04
0.035
0.03
0.025
0.02
0.015
0.01
0.005
0
Raman Intensity (A.U)
0 mM
7.5 mM
15 mM
30 mM
UPLC
I_{468}
I_{469}
I_{605}
I_{606}
R=0.70 R=0.72 R=0.74 R=0.73

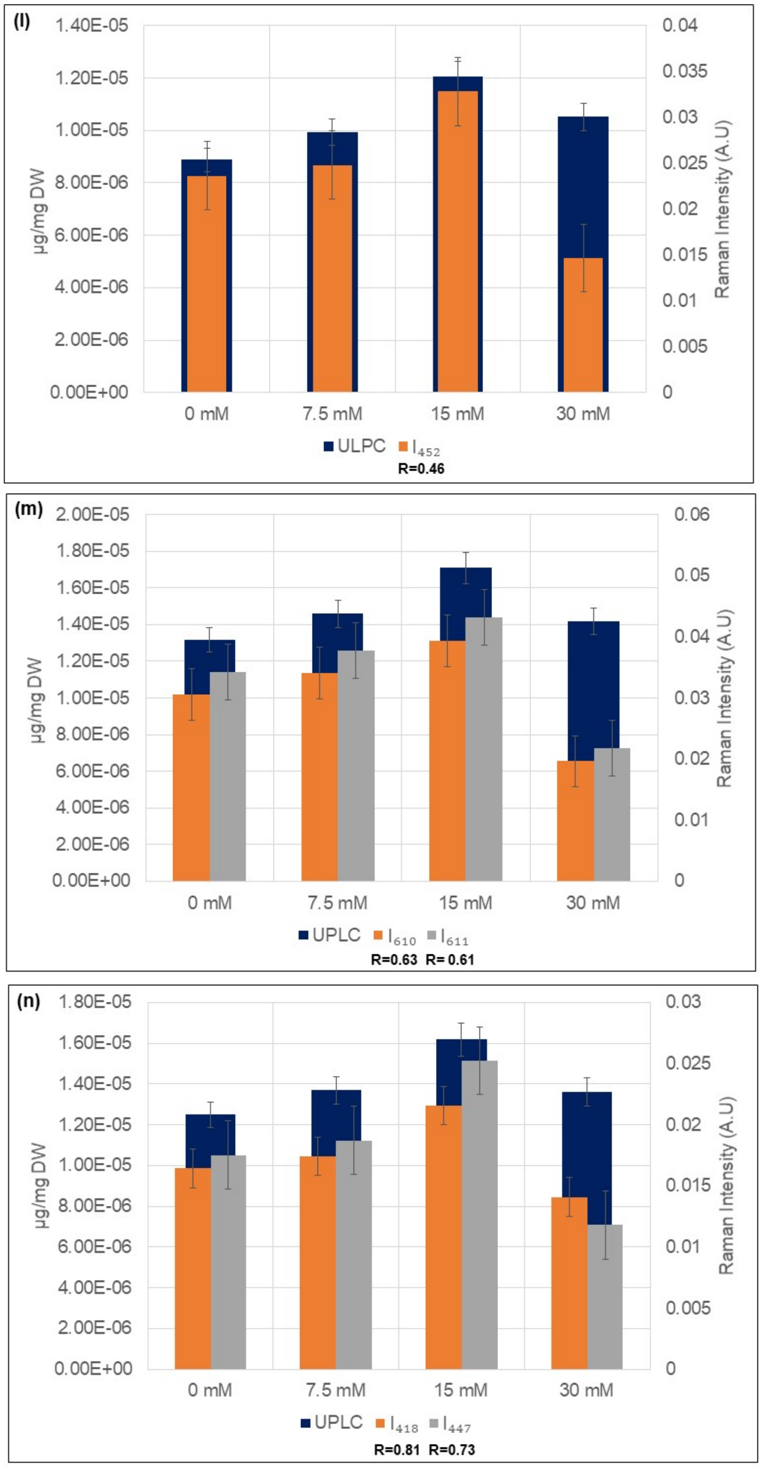
(l)
μg/mg DW
Raman Intensity (A.U)
0 mM
7.5 mM
15 mM
30 mM
ULPC
I_{452}
R=0.46
(m)
μg/mg DW
Raman Intensity (A.U)
0 mM
7.5 mM
15 mM
30 mM
UPLC
I_{610}
I_{611}
R=0.63 R= 0.61
(n)
μg/mg DW
Raman Intensity (A.U)
0 mM
7.5 mM
15 mM
30 mM
UPLC
I_{418}
I_{447}
R=0.81 R=0.73

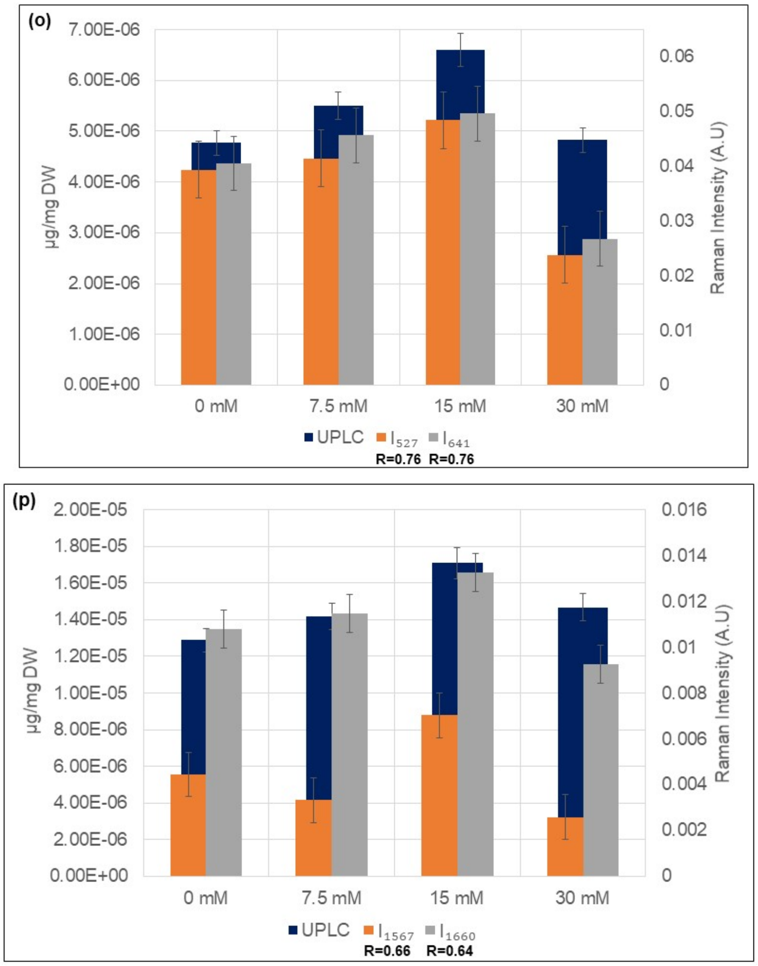

Figure S6. Correlation of Raman data with UPLC analysis of amino acids in the acetate study. (a) Alanine, (b) Arginine, (c) Aspartate/Asparagine, (d) Glutamate, (e) Glycine, (f) Histidine, (g) Isoleucine, (h) Leucine, (i) Lysine, (j) Methionine, (k) Phenylalanine, (l) Proline, (m) Serine, (n) Threonine, (o) Tyrosine, (p) Valine. Correlation coefficients (R) for each Raman band are represented.

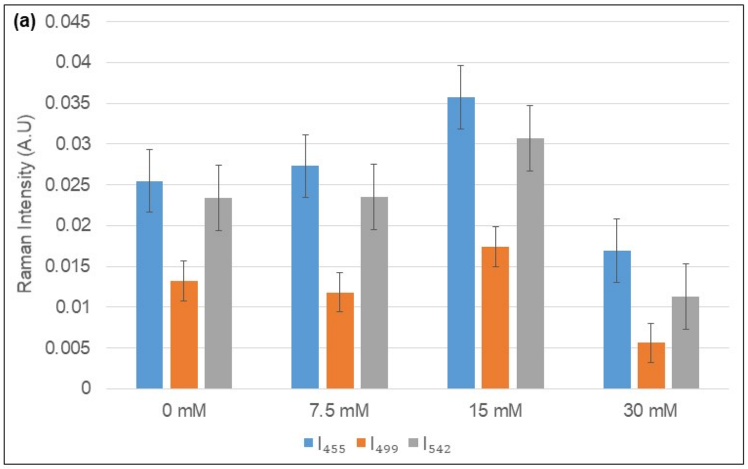

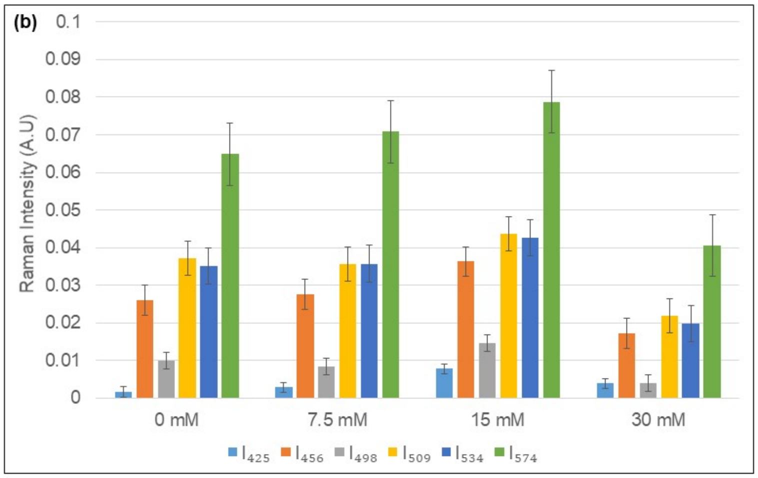

Figure S7. Acetate study: Raman bands predictions of amino acids non-resolved with UPLC. (a) Cysteine predictions, (b) Tryptophan predictions.

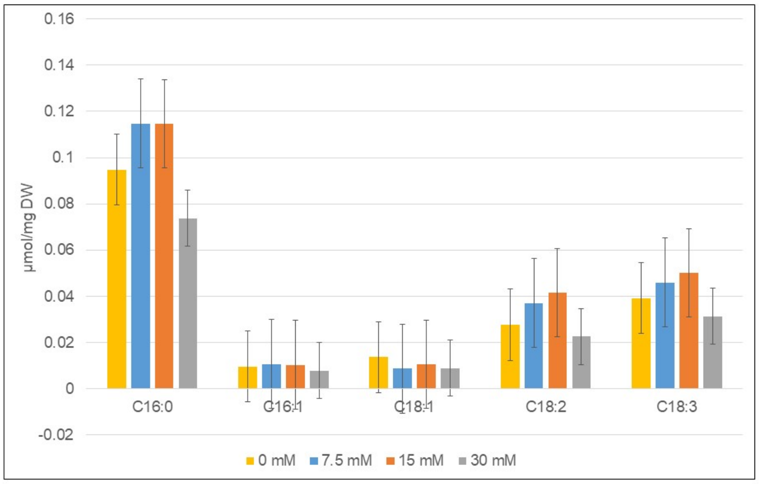

Figure S8. Levels of fatty acids for acetate study. Data represent means ± standard deviation (SD) of values from three independent experiments.

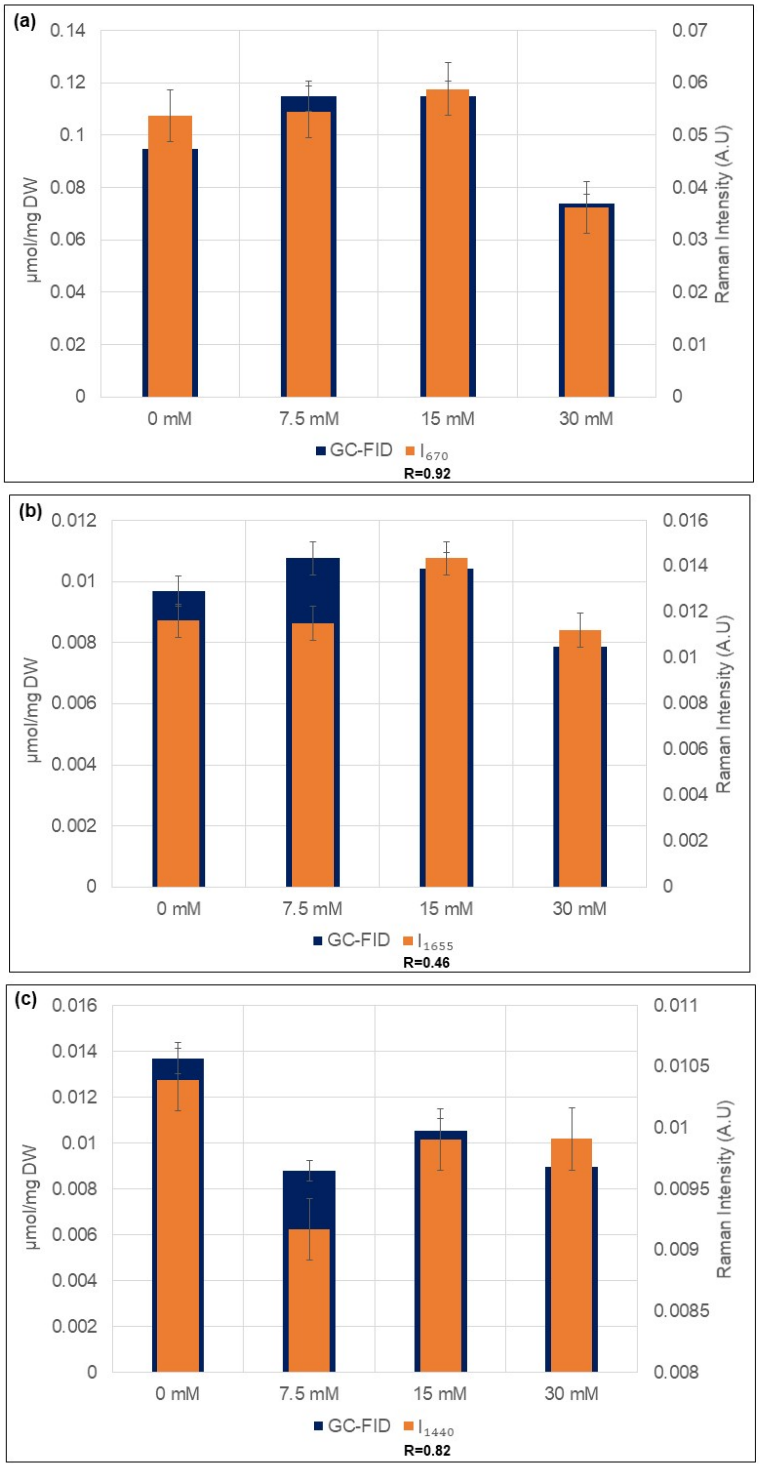
(a)
µmol/mg DW
Raman Intensity (A.U)
0 mM
7.5 mM
15 mM
30 mM
GC-FID
I670
R=0.92
(b)
µmol/mg DW
Raman Intensity (A.U)
0 mM
7.5 mM
15 mM
30 mM
GC-FID
I1655
R=0.46
(c)
µmol/mg DW
Raman Intensity (A.U)
0 mM
7.5 mM
15 mM
30 mM
GC-FID
I1440
R=0.82

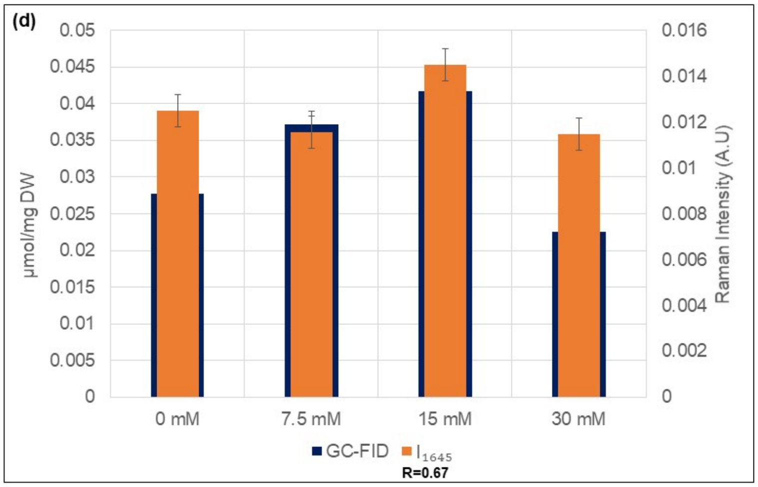

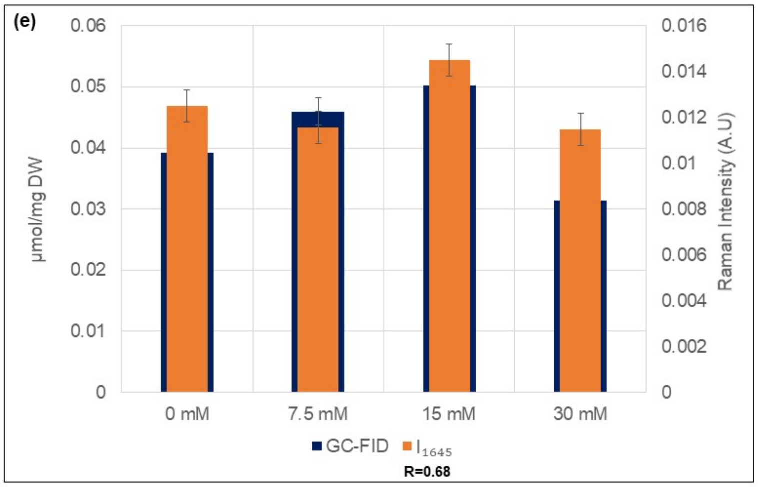

Figure S9. Correlation between Raman spectroscopy and GC-FID data of fatty acids for acetate study. (a) Palmitic acid, (b) Palmitoleic acid, (c) Oleic acid (d) Linoleic acid, (e) Linolenic acid. Correlation coefficients (R) for each Raman band are represented.

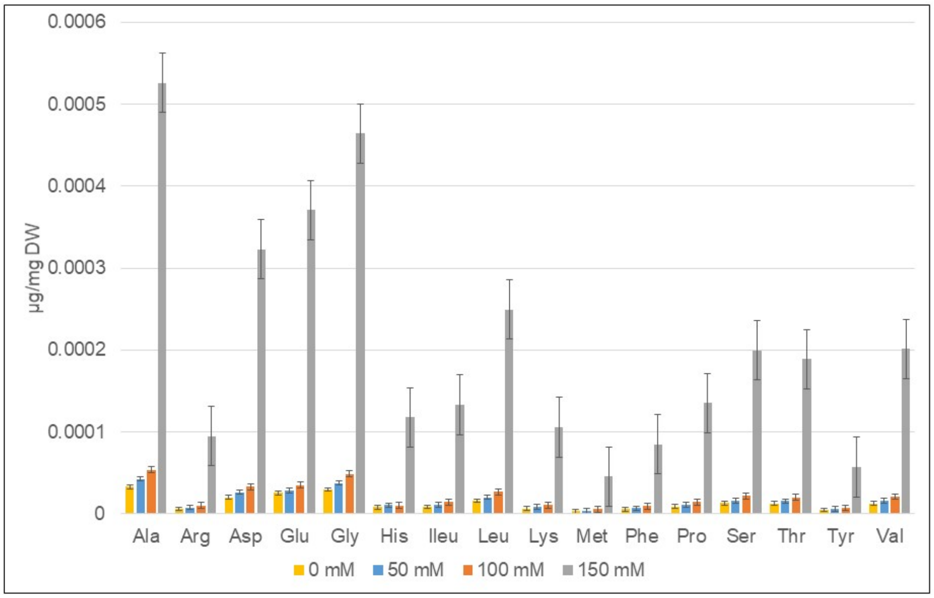

Figure S10. Amino acids analysis for NaCl study. Data represent means ± standard deviation (SD) of values from three independent experiments.

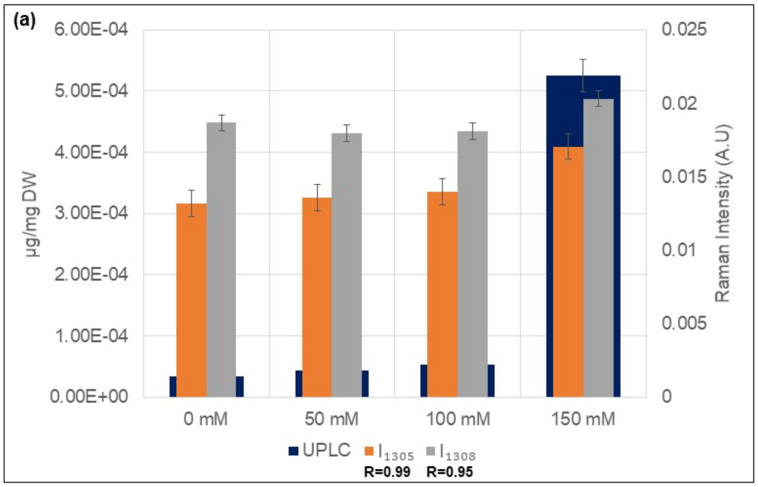

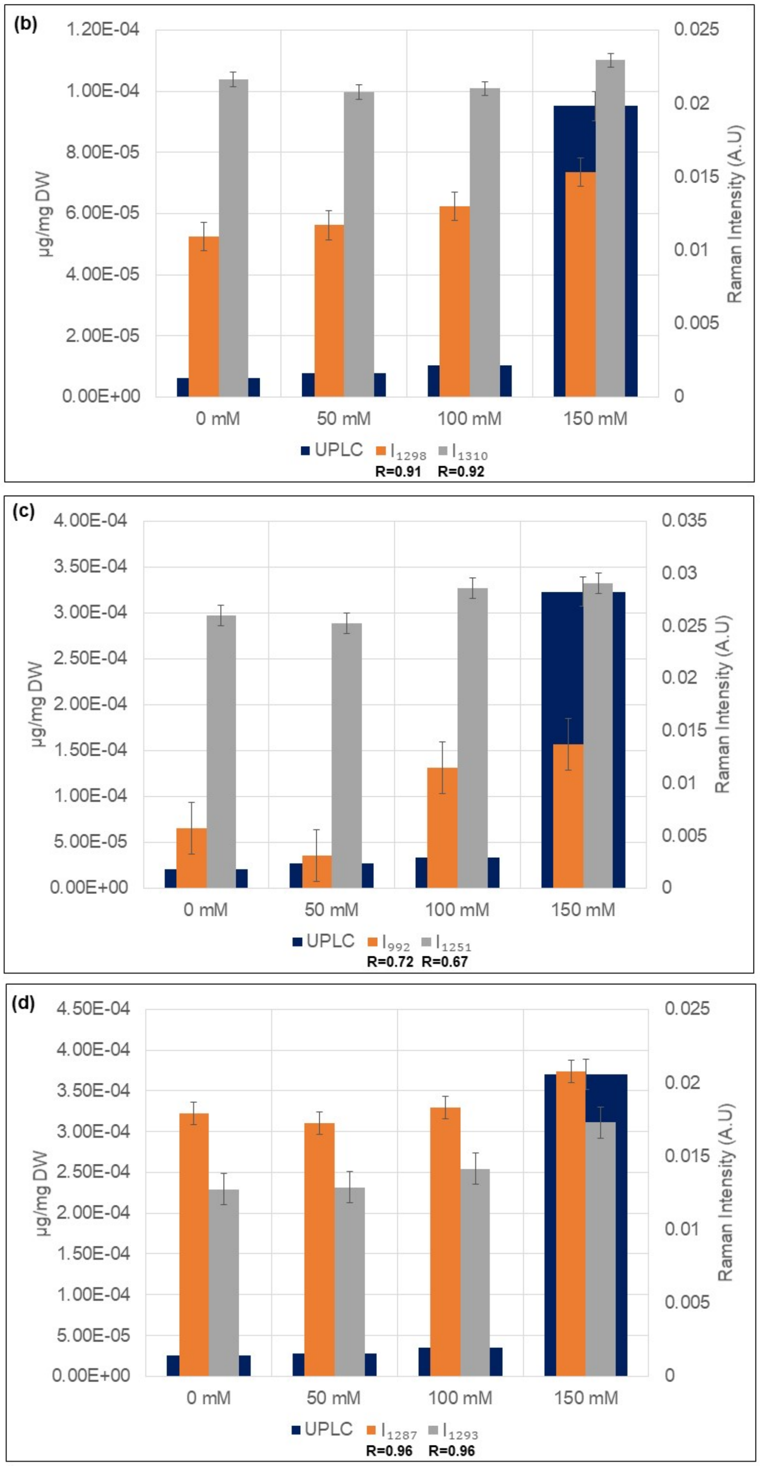
(b)
µg/mg DW
Raman Intensity (A.U)
1.20E-04
1.00E-04
8.00E-05
6.00E-05
4.00E-05
2.00E-05
0.00E+00
0.025
0.02
0.015
0.01
0.005
0
0 mM
50 mM
100 mM
150 mM
UPLC
I_{1298}
I_{1310}
R=0.91
R=0.92
(c)
µg/mg DW
Raman Intensity (A.U)
4.00E-04
3.50E-04
3.00E-04
2.50E-04
2.00E-04
1.50E-04
1.00E-04
5.00E-05
0.00E+00
0.035
0.03
0.025
0.02
0.015
0.01
0.005
0
0 mM
50 mM
100 mM
150 mM
UPLC
I_{992}
I_{1251}
R=0.72
R=0.67
(d)
µg/mg DW
Raman Intensity (A.U)
4.50E-04
4.00E-04
3.50E-04
3.00E-04
2.50E-04
2.00E-04
1.50E-04
1.00E-04
5.00E-05
0.00E+00
0.025
0.02
0.015
0.01
0.005
0
0 mM
50 mM
100 mM
150 mM
UPLC
I_{1287}
I_{1293}
R=0.96
R=0.96

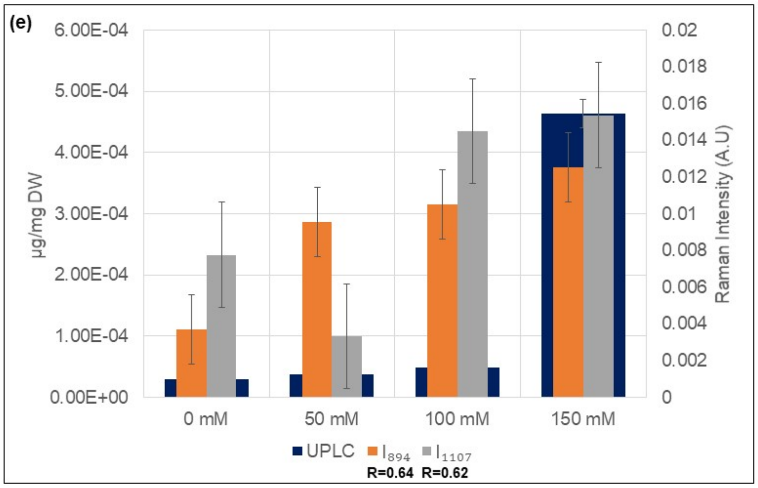
(e)
µg/mg DW
Raman Intensity (A.U)
0 mM
50 mM
100 mM
150 mM
UPLC
I_{894}
I_{1107}
R=0.64 R=0.62

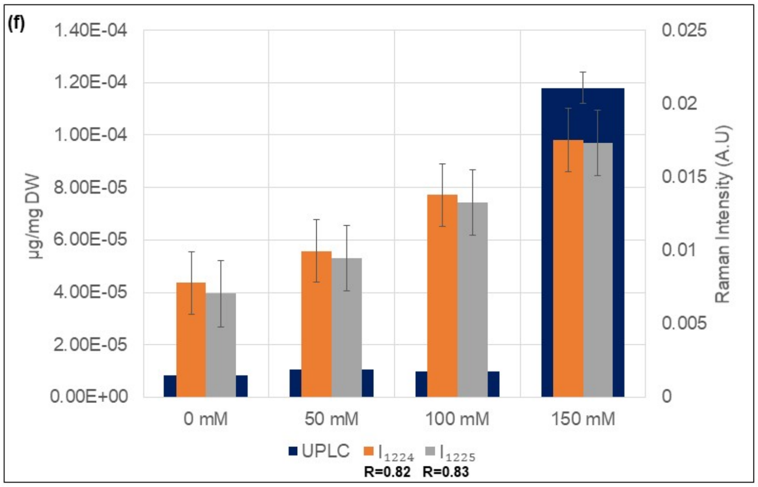
(f)
µg/mg DW
Raman Intensity (A.U)
0 mM
50 mM
100 mM
150 mM
UPLC
I_{1224}
I_{1225}
R=0.82 R=0.83

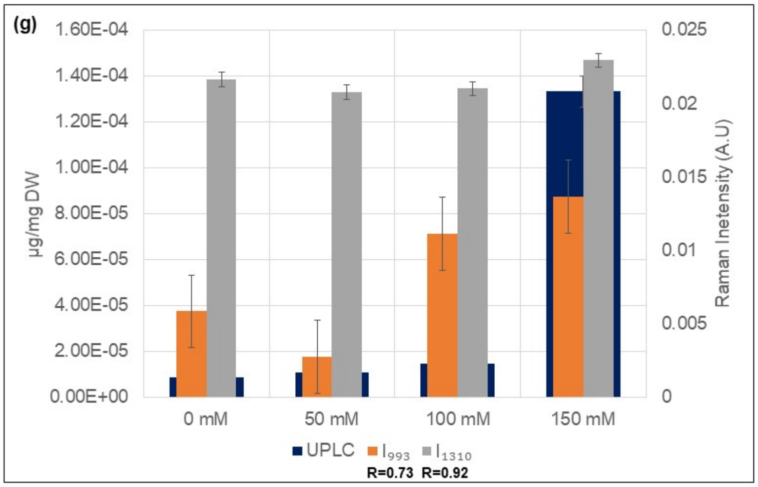
(g)
µg/mg DW
Raman Inetensity (A.U)
0 mM
50 mM
100 mM
150 mM
UPLC
I_{993}
I_{1310}
R=0.73 R=0.92

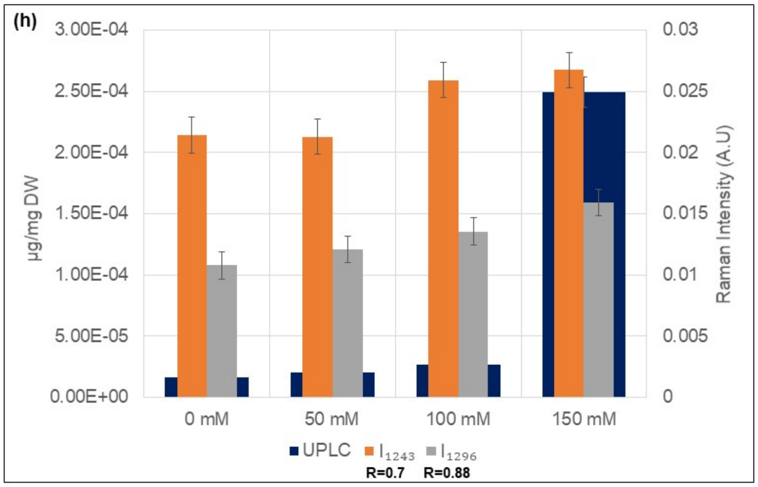
(h)
µg/mg DW
Raman Intensity (A.U)
3.00E-04
2.50E-04
2.00E-04
1.50E-04
1.00E-04
5.00E-05
0.00E+00
0.03
0.025
0.02
0.015
0.01
0.005
0
0 mM
50 mM
100 mM
150 mM
UPLC
I_{1243}
I_{1296}
R=0.7
R=0.88

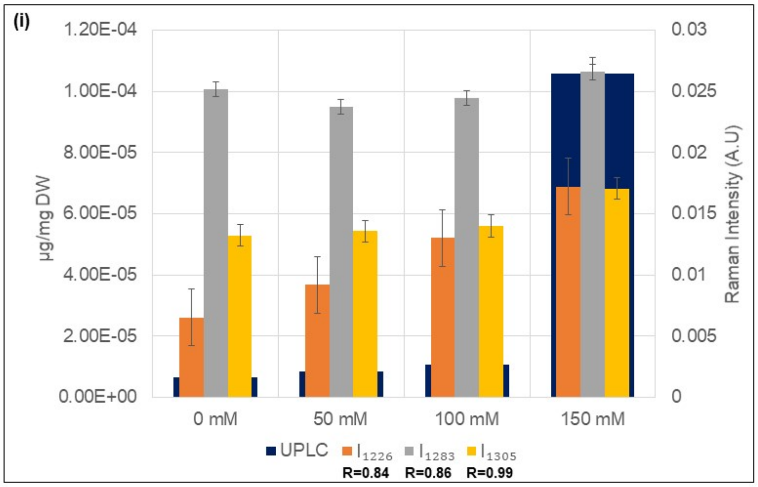
(i)
µg/mg DW
Raman Intensity (A.U)
1.20E-04
1.00E-04
8.00E-05
6.00E-05
4.00E-05
2.00E-05
0.00E+00
0.03
0.025
0.02
0.015
0.01
0.005
0
0 mM
50 mM
100 mM
150 mM
UPLC
I_{1226}
I_{1283}
I_{1305}
R=0.84
R=0.86
R=0.99

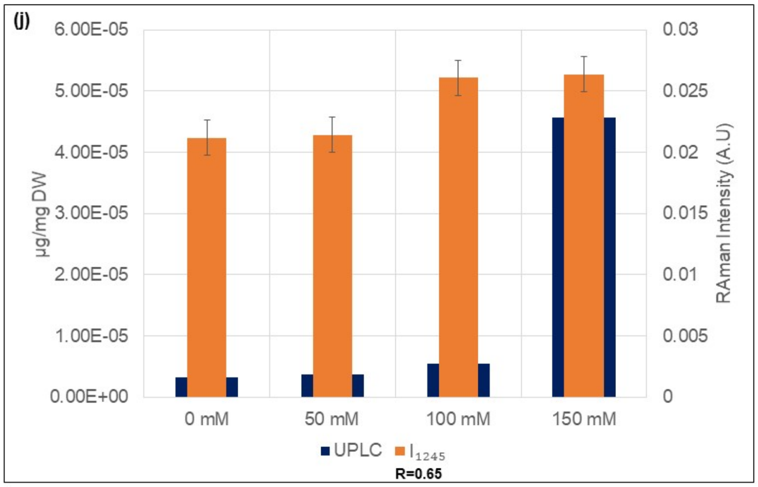
(j)
µg/mg DW
RAman Intensity (A.U)
6.00E-05
5.00E-05
4.00E-05
3.00E-05
2.00E-05
1.00E-05
0.00E+00
0.03
0.025
0.02
0.015
0.01
0.005
0
0 mM
50 mM
100 mM
150 mM
UPLC
I_{1245}
R=0.65

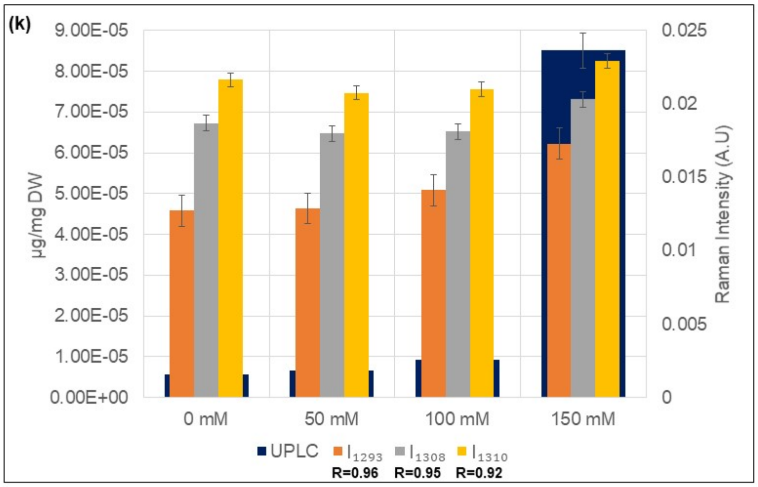
(k)
9.00E-05
8.00E-05
7.00E-05
6.00E-05
5.00E-05
4.00E-05
3.00E-05
2.00E-05
1.00E-05
0.00E+00
µg/mg DW
0.025
0.02
0.015
0.01
0.005
0
Raman Intensity (A.U)
0 mM
50 mM
100 mM
150 mM
UPLC
I1293
I1308
I1310
R=0.96 R=0.95 R=0.92

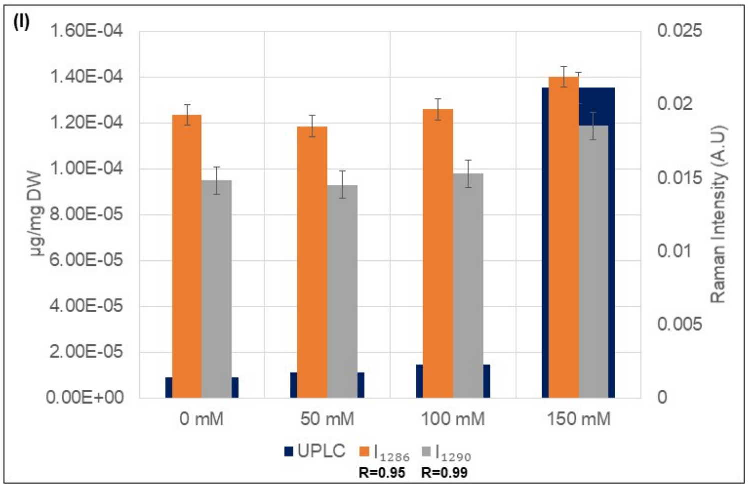
(l)
1.60E-04
1.40E-04
1.20E-04
1.00E-04
8.00E-05
6.00E-05
4.00E-05
2.00E-05
0.00E+00
µg/mg DW
0.025
0.02
0.015
0.01
0.005
0
Raman Intensity (A.U)
0 mM
50 mM
100 mM
150 mM
UPLC
I1286
I1290
R=0.95 R=0.99

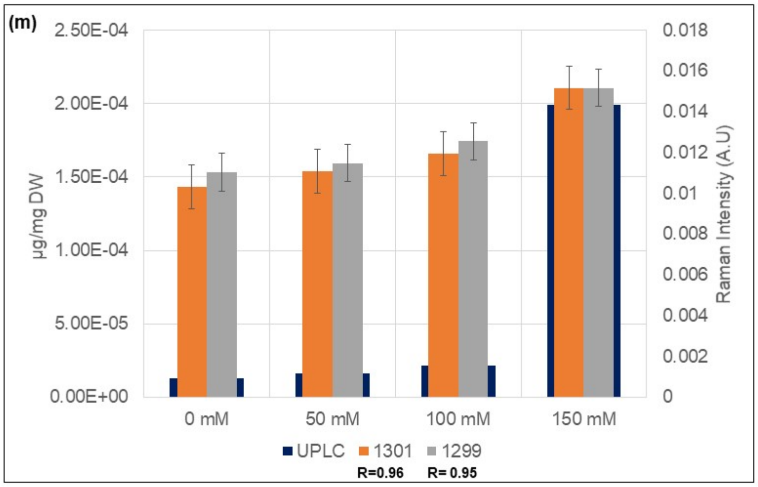
(m)
2.50E-04
2.00E-04
1.50E-04
1.00E-04
5.00E-05
0.00E+00
µg/mg DW
0.018
0.016
0.014
0.012
0.01
0.008
0.006
0.004
0.002
0
Raman Intensity (A.U)
0 mM
50 mM
100 mM
150 mM
UPLC
1301
1299
R=0.96 R= 0.95

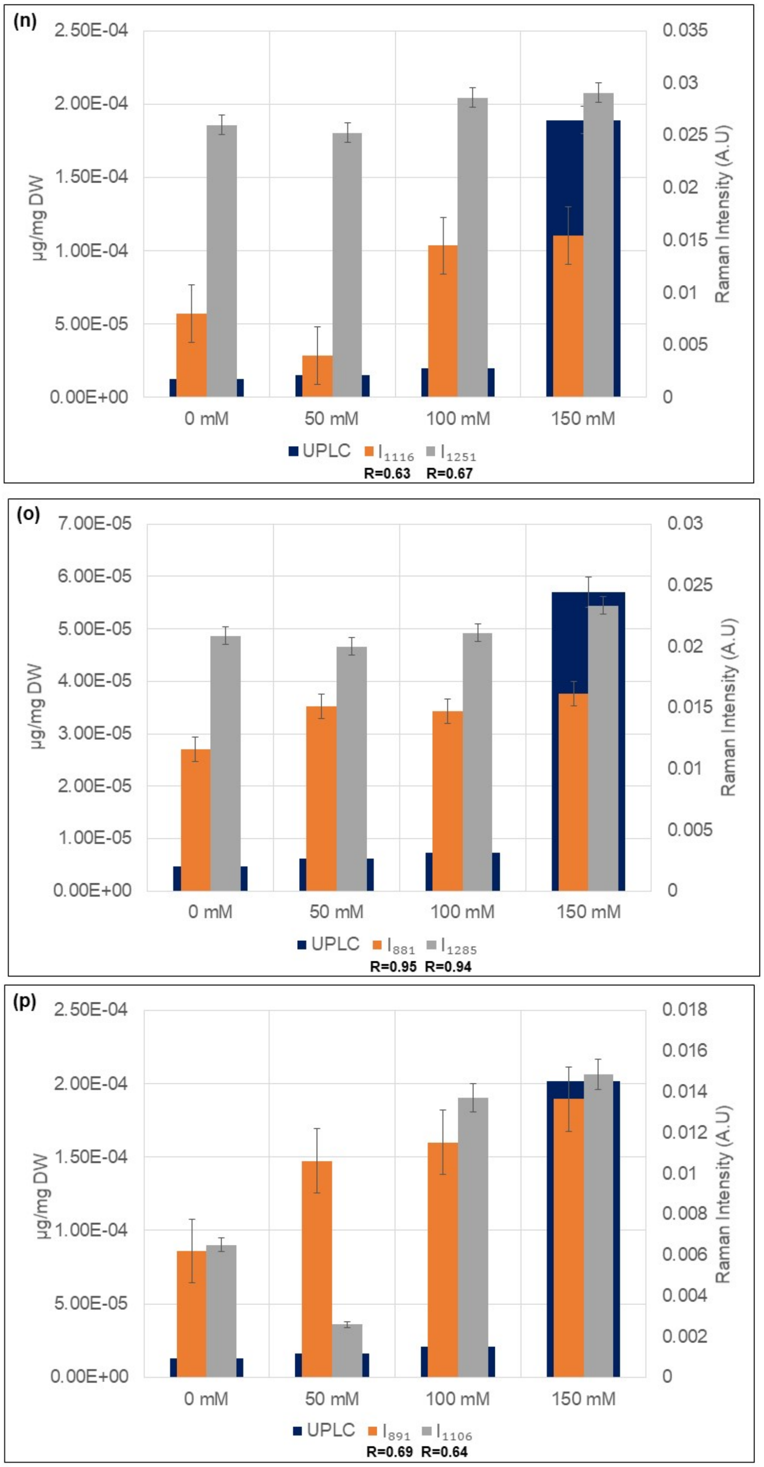

Figure S11. Correlation of Raman data with UPLC analysis of amino acids in the NaCl study. (a) Alanine, (b) Arginine, (c) Aspartate/Asparagine, (d) Glutamate, (e) Glycine, (f) Histidine, (g) Isoleucine, (h) Leucine, (i) Lysine, (j) Methionine, (k) Phenylalanine, (l) Proline, (m) Serine, (n) Threonine, (o) Tyrosine, (p) Valine. Correlation coefficients (R) for each Raman band are represented.

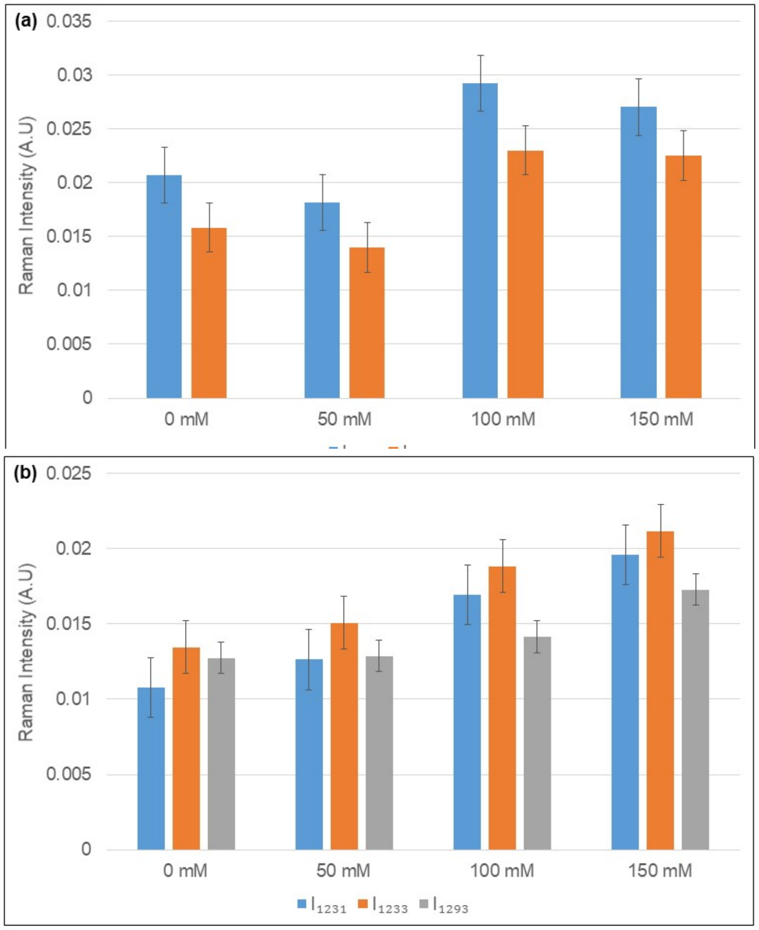

Figure S12. NaCl study: Raman bands predictions of amino acids non-resolved with UPLC. (a) Cysteine predictions, (b) Tryptophan predictions.

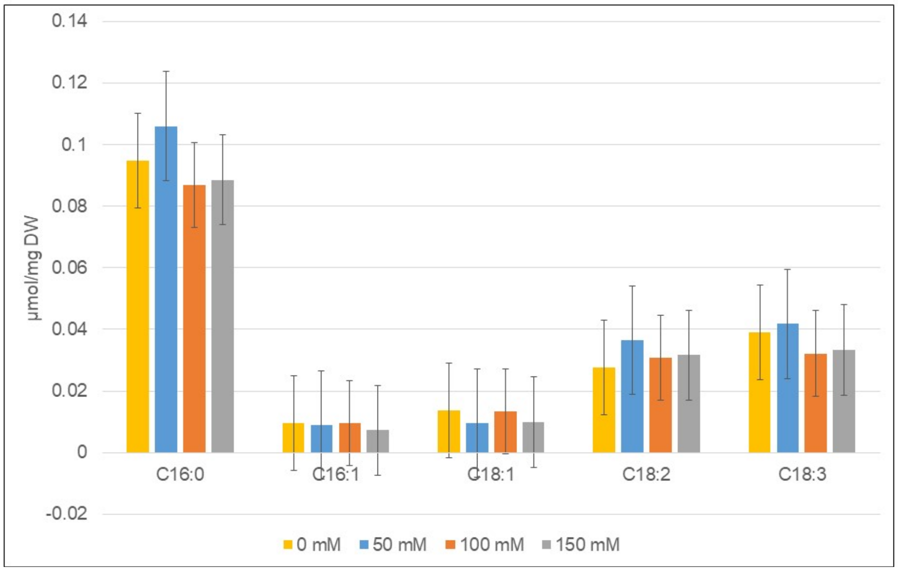

Figure S13. Levels of fatty acids for NaCl study. Data represent means ± standard deviation (SD) of values from three independent experiments.

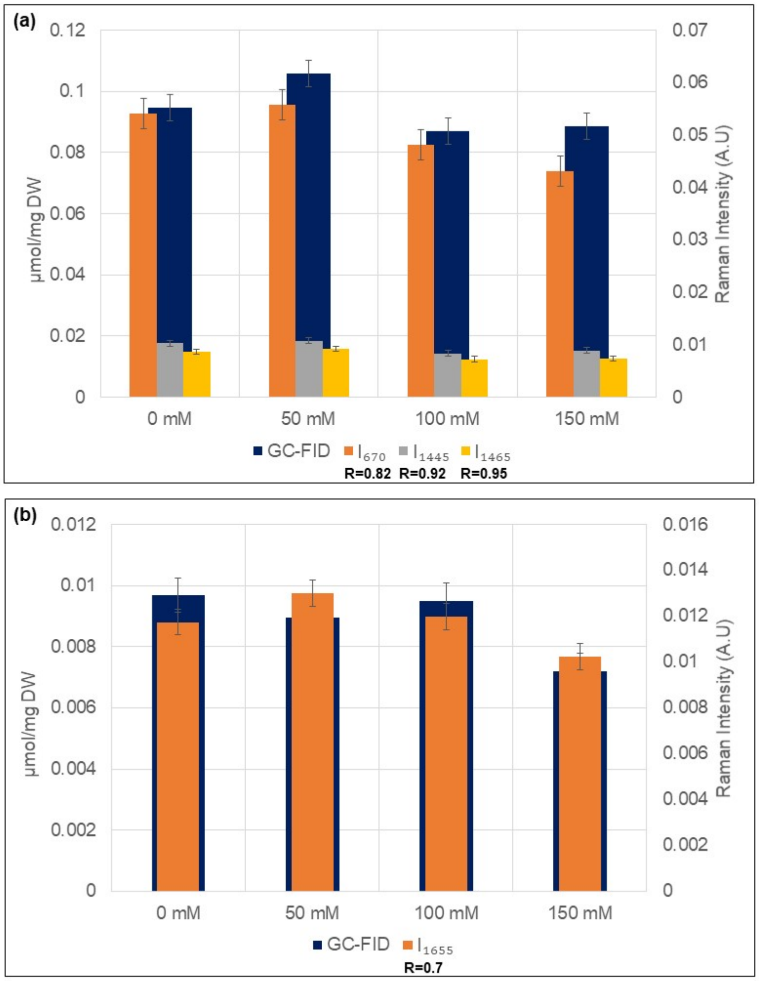

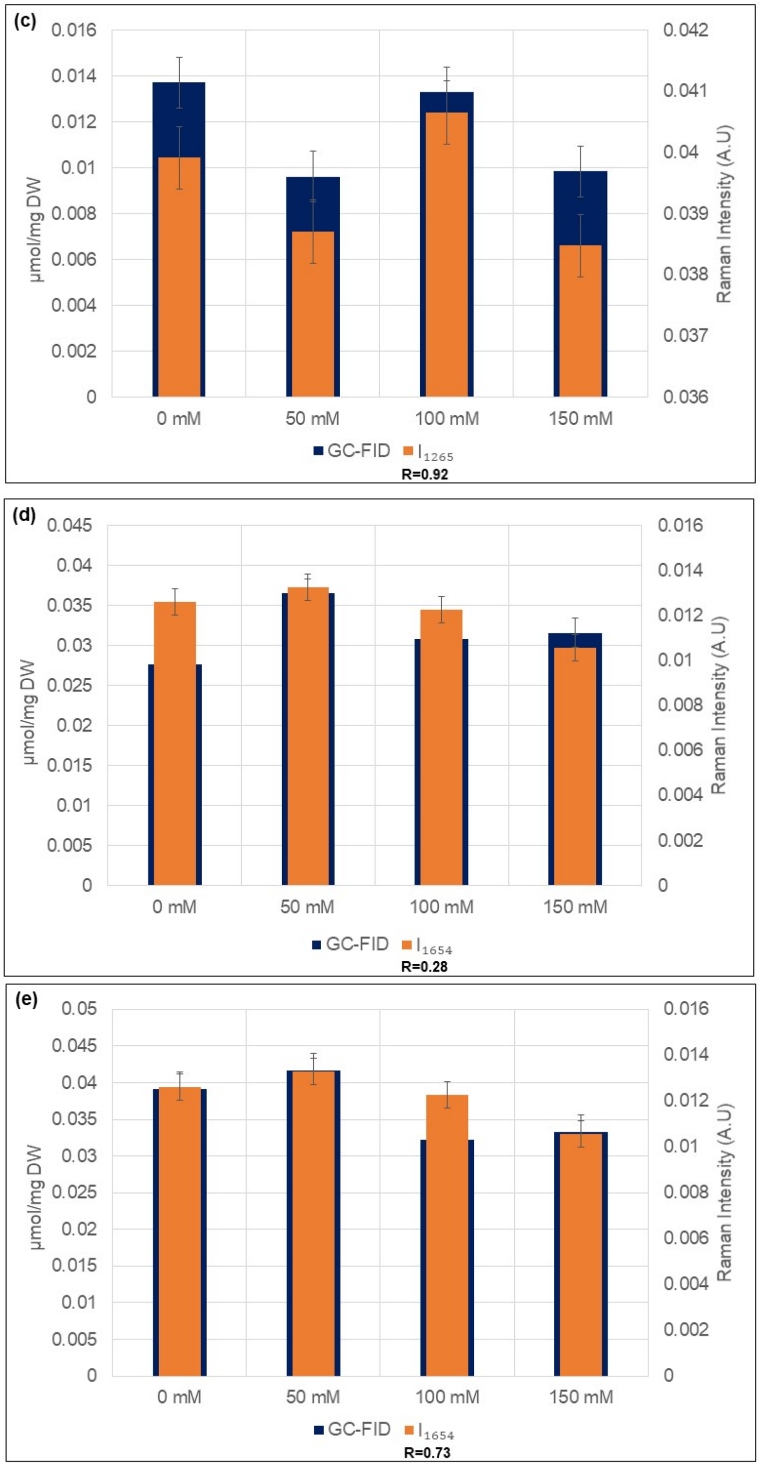

Figure S14. Correlation between Raman spectroscopy and GC-FID data of fatty acids for the NaCl study. (a) Palmitic acid, (b) Palmitoleic acid, (c) Oleic acid (d) Linoleic acid, (e) Linolenic acid. Correlation coefficients (R) for each Raman band are represented.

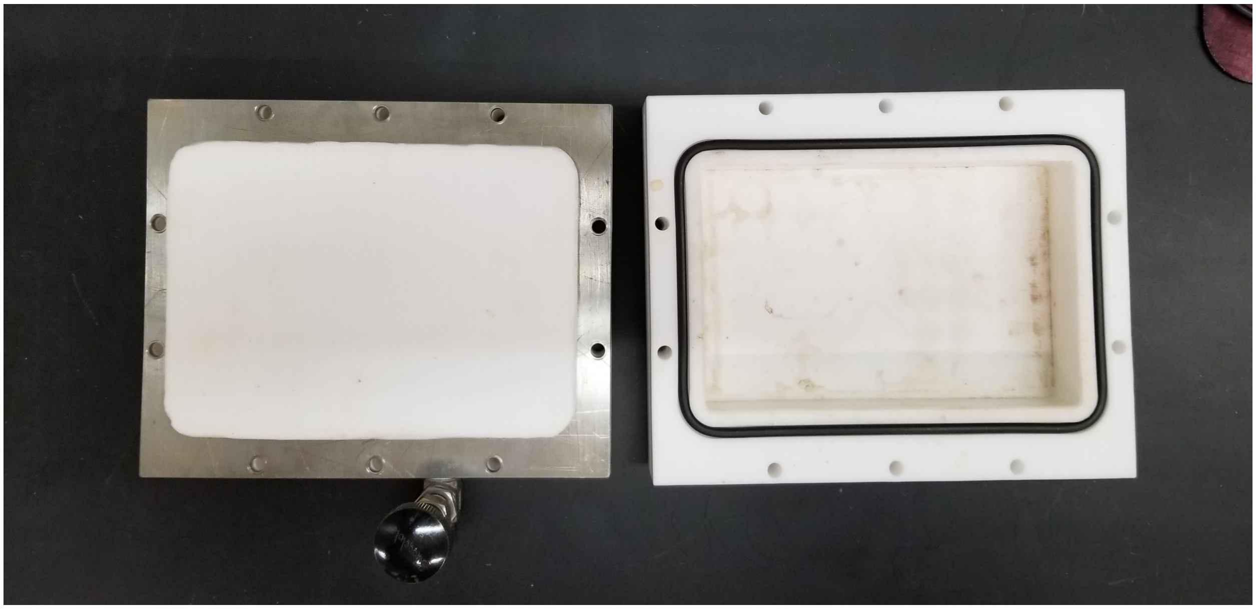

Figure S15. The fabricated teflon hydrolysis chamber.

Table S1. All Raman bands cited and tested for amino acids and fatty acids.

**Acetate Study**		
**Biomolecules**	**Raman bands (cm^{-1}) and Correlation Coefficient (R)**	**References**
Ala	480 (R = 0.45), 653 (R = 0.43)	(1,2)
Arg	1713 (R = 0.87)	(1)
Asp/Asn	1695 (R = 0.97)	(2)
Cys*	455, 499, 542	(2)
Glu/Gln	1462 (R = 0.87), 1624 (R = 0.73)	(1,2)
Gly	496 (R = 0.71), 1568 (R = 0.73), 1674 (R = 0.84)	(2)

His	422 (R = 0.79)	(1)
Ile	557 (R = 0.63)	(2)
Leu	1583 (R = 0.49)	(2)
Lys	1570 (R = 0.5)	(2)
Met	645 (R = 0.59)	(2)
Phe	468 (R = 0.70), 469 (R = 0.72), 0.74 (R = 605), 606 (R = 0.73)	(1,2)
Pro	452 (R = 0.46)	(1)
Ser	610 (R = 0.63), 611 (R = 0.61)	(1,2)
Thr	418 (R = 0.81), 447 (R = 0.73)	(2)
Trp*	425, 456, 498, 509, 509, 574	(1)
Tyr	527 (R = 0.76), 641 (R = 0.76)	(1,2)
Val	1567 (R = 0.66), 1660 (R = 0.64)	(1)
Palmitic acid (C16:0)	670 (R = 0.92)	(1)
Palmitoleic acid (C16:1)	1655 (R = 0.46)	(3)
Oleic acid (C18:1)	1440 (R = 0.82)	(1)
Linoleic acid (C18:2)	1645 (R = 0.67)	(3)
Linolenic acid (C18:3)	1645 (R = 0.68)	(3)
**Sodium chloride Study**		

Biomolecules	Raman bands (cm^{-1}) and Correlation Coefficient (R)	References
Ala	1305 (R = 0.99), 1308 (R = 0.95)	(1,2)
Arg	1298 (R = 0.91), 1310 (R = 0.92)	(1,2)
Asp/Asn	992 (R = 0.72), 1251 (R = 0.67)	(2)
Cys*	1041, 1092	(2)
Glu/Gln	1287 (R = 0.96), 1293 (R = 0.96)	(1,2)
Gly	894 (R =0.64), 1107 (R = 0.62)	(1,2)
His	1224 (R = 0.82), 1225 (R = 0.83)	(1,2)
Ile	993 (R = 0.73), 1310 (R = 0.92)	(2)
Leu	1243 (R = 0.7), 1296 (R = 0.88)	(2)
Lys	1226 (R = 0.84), 1283 (R = 0.86), 1305 (R = 0.99)	(2)
Met	1245 (R = 0.65)	(2)
Phe	1293 (R = 0.96), 1308 (R = 0.95), 1310 (R = 0.92)	(1,2)
Pro	1286 (R = 0.95), 1290 (R = 0.99)	(1)
Ser	1299 (R = 0.95), 1301 (R = 0.96)	(1,2)
Thr	1116 (R = 0.63), 1251 (R = 0.67)	(2)
Trp*	1231, 1233, 1293	(1,2)
Tyr	881 (R = 0.95), 1285 (R = 0.94)	(2)

Val	891 (R = 0.69), 1106 (R = 0.64)	(1)
Palmitic acid (C16:0)	670 (R = 0.82), 1445 (R = 0.92), 1465 (R = 0.95)	(1)
Palmitoleic acid (C16:1)	1655 (R = 0.7)	(3)
Oleic acid (C18:1)	1265 (R = 0.92)	(1)
Linoleic acid (C18:2)	1654 (R = 0.28)	(3)
Linolenic acid (C18:3)	1654 (R = 0.73)	(3)

* Values for Cys and Trp could not be obtained by the UPLC method used in this research. Only Raman predictions are provided.

References

1. De Gelder J, De Gussem K, Vandenabeele P, Moens L. Reference database of Raman spectra of biological molecules. J Raman Spectrosc. 2007;38(9):1133–47.
2. Zhu G, Zhu X, Fan Q, Wan X. Raman spectra of amino acids and their aqueous solutions. Spectrochim Acta Mol Biomol Spectrosc. 2011;78(3):1187–95.
3. Czamara K, Majzner K, Pacia MZ, Kochan K, Kaczor A, Baranska M. Raman spectroscopy of lipids: a review. J Raman Spectrosc. 2015;46(1):4–20.
